# Functionally related transcripts have common RNA motifs for specific RNA-binding proteins in trypanosomes

**DOI:** 10.1186/1471-2199-9-107

**Published:** 2008-12-08

**Authors:** Griselda Noé, Javier G De Gaudenzi, Alberto C Frasch

**Affiliations:** 1 Instituto de Investigaciones Biotecnológicas-Instituto Tecnológico Chascomús, UNSAM-CONICET, Av. Gral. Paz 5445, INTI, Edificio 24, 1650 San Martín, Provincia de Buenos Aires, Argentina

## Abstract

**Background:**

Trypanosomes mostly control gene expression by post-transcriptional events such as modulation of mRNA stability and translational efficiency. These mechanisms involve RNA-binding proteins (RBPs), which associate with transcripts to form messenger ribonucleoprotein (mRNP) complexes.

**Results:**

In this study, we report the identification of mRNA targets for *Trypanosoma cruzi *U-rich RBP 1 (*Tc*UBP1) and *T. cruzi *RBP 3 (*Tc*RBP3), two phylogenetically conserved proteins among Kinetoplastids. Co-immunoprecipitated RBP-associated RNAs were extracted from mRNP complexes and binding of RBPs to several targets was confirmed by independent experimental assays. Analysis of target transcript sequences allowed the identification of different signature RNA motifs for each protein. *Cis*-elements for RBP binding have a stem-loop structure of 30–35 bases and are more frequently represented in the 3'-untranslated region (UTR) of mRNAs. Insertion of the correctly folded RNA elements to a non-specific mRNA rendered it into a target transcript, whereas substitution of the RNA elements abolished RBP interaction. In addition, RBPs competed for RNA-binding sites in accordance with the distribution of different and overlapping motifs in the 3'-UTRs of common mRNAs.

**Conclusion:**

Functionally related transcripts were preferentially associated with a given RBP; *Tc*UBP1 targets were enriched in genes encoding proteins involved in metabolism, whereas ribosomal protein-encoding transcripts were the largest group within *Tc*RBP3 targets. Together, these results suggest coordinated control of different mRNA subsets at the post-transcriptional level by specific RBPs.

## Background

*Trypanosoma cruzi*, a protozoan parasite of the order Kinetoplastida, is the causative agent of Chagas disease in Latin America. This protist, like the African trypanosome *Trypanosoma brucei*, has a complex life cycle and alternates between insect vectors and mammalian hosts. Being a single cell that suffers continuous environmental changes, *T. cruzi *needs to quickly regulate the expression of many genes to allow rapid adaptation (reviewed in references [1] and [2]). Such microorganisms control protein synthesis mostly by post-transcriptional mechanisms. Transcription in trypanosomes is polycistronic [3] and, in contrast to what occurs in bacterial operons, polycistronic units must be co-transcriptionally processed before translation [4], by coupled 5'-*trans*-splicing and 3'-polyadenylation events [5-7]. However, with a single exception [8], no classical promoters have been identified in trypanosomes, and thus there is no evidence for controlled transcriptional initiation of genes through modulation of RNA polymerase II activity [9]. Given these peculiarities, trypanosomes represent an interesting model for studies on mechanisms of post-transcriptional regulation of gene expression [3,10], in which mRNA degradation/stabilization is the main control feature. Active deadenylation systems have been found in trypanosome cells [11,12]. After removal of the poly(A) tail and the 5'-cap, the mRNA can be degraded from both ends by XRN1-related exoribonucleases (5'-3' direction) and the exosome (3'-5' direction) (reviewed in reference [13]; see also references [14] and [15]). RNA interference is also involved in gene-silencing phenomena in some species of the Trypanosomatidae family [16,17].

Mature transcripts contain regulatory motifs located in the 5'- and 3'-untranslated regions (UTRs) that modulate transcript abundance by specific interaction with RNA-binding proteins (RBPs). These *cis*-elements are involved in the control of mRNA transport, stability, and translation efficiency [18,19]. Several RBPs form, together with mRNAs, a network of messenger-ribonucleoprotein (mRNP) complexes directing post-transcriptional regulation in response to diverse stimuli [20]. An important class of these factors contains an RNA-binding domain called RNA-recognition motif (RRM) [21].

The genome sequencing projects of three trypanosomatids (*T. cruzi*, *T. brucei *and *Leishmania major*) was completed in 2005 [22-24], providing crucial data for study of gene content and genome organization. Specifically, a superfamily of more than 100 RRM-type proteins was discovered in the *T. cruzi *genome [25]. Some are involved in alternative splicing processes, mRNA stabilization/degradation, polyadenylation, or translational control. However, the majority do not have clear homologs in other species, even though they are highly conserved in Kinetoplastids. Among them, a family containing about 20 members, shares a common RRM sequence but contain different auxiliary domains [26]. One member of this protein family is *T. cruzi *U-rich RBP 1 (*Tc*UBP1) [27], a single RRM domain cytoplasmic RBP with a characteristic βαββαβ-fold flanked by N-terminal Gln-rich and C-terminal Gly-Gln-rich extensions that are likely involved in protein-protein interactions [28]. This protein shares almost the same RRM sequence (99% identity) with a second RBP family member termed *Tc*UBP2. Previous work from our laboratory has shown that both proteins can form a complex with poly(A)-binding protein PABP1 at the 3'-UTR of *mucin *transcripts, producing selective destabilization of such mRNAs [27]. *T. cruzi *RBP 3 (*Tc*RBP3) is a third RBP family member that shares less than 60% identity with the RRM domain of *Tc*UBP1 [26]; this is reflected in different *in vitro *binding characteristics (see below). *T. brucei *has homologous RBPs, two of these, termed *Tb*UBP1 and *Tb*UBP2, are involved in stabilization of *cyclin F *box mRNA and a transmembrane protein gene family [29,30].

To understand the possible roles of trypanosome RBPs, it is necessary to identify mRNA targets whose half-lives or activities are modulated by interactions with the RBPs. *Tc*UBP1 has been reported to bind to 3'-UTR sequences encompassing AU-rich elements (AREs) with AUUUA, AUUUUA, and AUUUUUA motifs, and showed specificity for poly(U) and poly(G) homoribopolymers. *Tc*RBP3 displayed a different homoribopolymer-binding pattern, showing specificity for poly(A), poly(C), and poly(G) [26]. In this study, we systematically identified cellular targets for *Tc*UBP1 and *Tc*RBP3 and report on structural RNA elements conserved in each group of transcripts. These signature RNA motifs were successfully used to predict putative *Tc*UBP1 and *Tc*RBP3 target mRNAs from trypanosome databases.

## Results

### Identification of *Tc*UBP1 and *Tc*RBP3 mRNA targets

**Figure 1:**
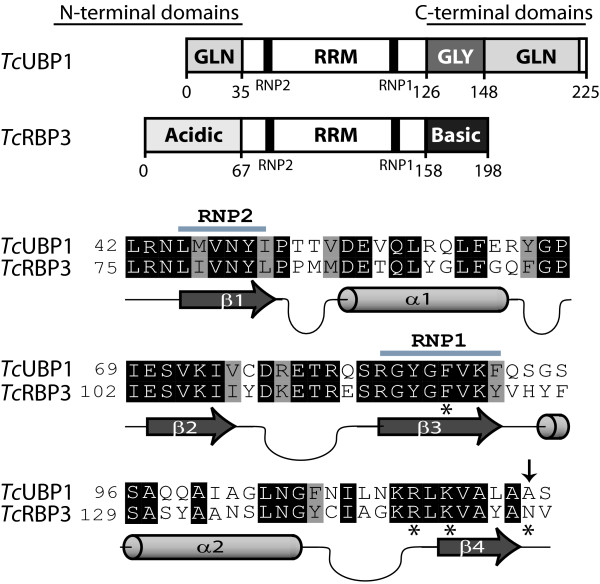
**Pair wise alignment of *Tc*UBP1 and *Tc*RBP3**. Proteins domains of *Tc*UBP1 and *Tc*RBP3 are indicated. A comparison of their RNA-binding domains was performed using ClustalW [70]. Conserved residues are shown in black and RNP1 and RNP2 motifs are underlined. * residues involved in RNA recognition, the amino acid that differs is indicated with an arrow.

**Figure 2:**
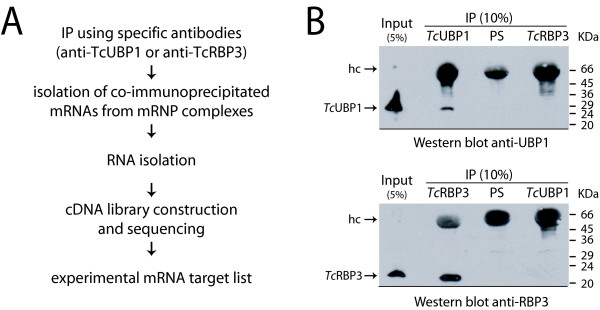
**Scheme of experimental approach and immunoprecipitation experiments**. A) Scheme of the experimental approach. For IP, cytosolic cell-free extracts from epimastigotes (Input) were incubated with preimmune serum and anti-*Tc*UBP1 or anti-*Tc*RBP3 antibodies. RNA extracted from immunoprecipitated mRNP complexes was used to perform RT-PCR and to construct a cDNA library. B) Detection of proteins was done by Western blot employing specific antibodies. The position of the bands corresponding to each protein and to the heavy chain (hc) of antibodies is indicated with an arrow on the left side of the panel.

A comparative RNA-protein interaction analysis was performed using mRNP immunopurification assays, with the aid of specific antibodies raised against each RRM-type protein, *Tc*UBP1 and *Tc*RBP3. These two RBPs show distinct amino acid compositions in their RRM regions (Fig. 1). In particular, the sequences demonstrate differences within the loops of the RNA-binding domain spatial structure, which has been shown to confer specificity for RNA-binding in other proteins [31,32]. In addition, both proteins differ at one amino acid position (Asn^153 ^in *Tc*RBP3 vs Ala^120 ^in *Tc*UBP1), which is one of the four *Tc*UBP1 residues (Phe^88^, Arg^113^, Lys^115 ^and Ala^120^) predicted to be involved in RNA recognition [28]. To determine if this change might influence the nature of transcripts with which the RBPs can associate, cytosolic cell-free extracts of *T. cruzi *epimastigotes prepared under conditions that preserved mRNA-protein interactions were incubated with either anti-*Tc*UBP1 or anti-*Tc*RBP3 antibodies. The RNA extracted from mRNP complexes was reverse transcribed using an oligo(dT) primer, amplified by PCR, and used to construct a library of transcripts associated with each protein (see Fig. 2A for the experimental approach and Methods for details). A fraction (10%) of the immunoprecipitation (IP) material was used to analyze proteins by Western-blot assays. As shown in Figure 2B, each antibody can specifically detect unique bands of the expected sizes in protein extracts (Input). Furthermore, *Tc*UBP1 was specifically immunoprecipitated by anti-*Tc*UBP1 antibody, whereas *Tc*RBP3 was found in the *Tc*RBP3-IP material. In contrast, both proteins were undetectable in the reciprocal IP assay or when the IP was performed using preimmune serum.

Our analysis revealed that, among the identified *Tc*UBP1-bound mRNAs, 24 sequences shared significant similarity (>95%) to known genes and 10 to hypothetical protein transcripts. A large proportion of the transcripts identified code for proteins involved in metabolism or glycoproteins. The list of *Tc*UBP1 target hits is shown in Table 1. Transcripts found in *Tc*RBP3 IP material comprise 3 hypothetical proteins and 21 annotated mRNAs, including 12 different ribosomal protein-encoding (RPC) genes of large and small subunit proteins (see Table 2 for a complete list of RBP3-bound RNAs). RPC transcripts are abundant cellular mRNAs that may co-precipitate non-specifically; their detection might thus be attributable to non-specific association. However, these transcripts were not immunoprecipitated by preimmune serum or an unrelated RBP antibody (see Additional File 1), indicating that the RPC genes identified are *bona fide *targets. Sequences reported as Expressed Sequence Tags (ESTs) were also present in both IPs. In addition, common targets such as *28S *ribosomal RNA (rRNA) (L22334), *18S *rRNA (CF243364), *Hypothetical protein 1 *(*HYPO1*) (Tc00.1047053506811.120) and mitochondrial transcripts [*ATPase subunit 6 (ATPase) *(DQ343645 Region: 9998-10333) and *Cytochrome oxidase subunit III *(*COIII*) (DQ343645 Region: 8384-8806)], were found in both UBP1- and RBP3-IPs (see below). A preimmune serum and anti-*Tc*Rpi antibody were used as controls and did not immunoprecipitate visible amounts of mRNA (see Additional File 1).

**Table 1:** Experimental *Tc*UBP1-associated RNAs.

Description	Transcript name	Systematic name or GenBank Acc. Num.
***mRNAs***		
D-alanyl-glycyl endopeptidase-like protein	*ENDO*	Tc00.1047053507715.10
Zinc finger protein 1	*ZFP1*	Tc00.1047053511511.6
Ribosomal protein L19	*RPL19*	Tc00.1047053508175.309
Pyruvate dehydrogenase E1 sub. beta	*PDH*	Tc00.1047053510091.80
Pyruvate dehydrogenase E1 sub. beta (TENS1250)	*PDH*	Tc00.1047053510421.320
Surface protease GP63	*GP63-1*	Tc00.1047053510747.40
Cytochrome b5	*CYTB5*	Tc00.1047053509395.100
Membrane-associated protein	*MAP*	Tc00.1047053507795.10
Fatty acid elongase	*FAE*	Tc00.1047053511245.150
*T. cruzi *85-kD surface antigen	*GP85*	Tc00.1047053506455.30
Amino acid transporter	*AAT*	Tc00.1047053511325.25
Myosin-like protein	*MYOLP*	Tc00.1047053511527.70
Endosomal trafficking protein RME-8 (TENS0888)	*RME-8*	Tc00.1047053511511.10
Mucin-like protein SMUG (clone 7.2)	*SMUG*	Tc00.1047053504539.20
Amastin	*AMAS*	Tc00.1047053506437.30
Mucin-like protein EMUC (clone e-1a4)	*EMUC*	Tc00.1047053503761.30
Cysteine proteinase (Cruzipain)	*CRUZ*	Tc00.1047053509429.320
Trans-sialidase*	*TRANS*	Tc00.1047053509495.30
Hypothetical protein 1	*HYPO1*	Tc00.1047053506811.120
Hypothetical protein 2	*HYPO2*	Tc00.1047053508015.40
Hypothetical protein 3	*HYPO3*	Tc00.1047053509297.20
Hypothetical protein 4	*HYPO4*	Tc00.1047053508175.90
Hypothetical protein 5	*HYPO5*	Tc00.1047053507085.120
Hypothetical protein 6*	*HYPO6*	Tc00.1047053506813.5
Hypothetical protein 7	*HYPO7*	Tc00.1047053511623.10
Hypothetical protein 8 (TENS1110)	*HYPO8*	Tc00.1047053507949.250
Hypothetical protein 9 (TENF0687) *	*HYPO9*	Tc00.1047053511623.20
Hypothetical protein 10 (TENU0658)	*HYPO10*	Tc00.1047053511911.90
		
***Expressed sequence tags (ESTs)***		
CB964273	*EST1*	CB964273
TEUF0191	*EST2*	AA433339
TEUF0210	*EST3*	AA676196
SA-7-4	*EST4*	BF299423
TENU3101*	*EST5*	AI075507
TENU3771	*EST6*	AI080913
		
***Ribosomal RNA***		
28S ribosomal RNA*	*28S *rRNA	L22334
18S ribosomal RNA (TENQ0845)	*18S *rRNA	CF243364
		
***Mitochondrial transcripts***		
ATPase subunit 6*	*ATPase 6*	DQ343645
Cytochrome oxidase subunit III*	COIII	DQ343645
Cytochrome oxidase subunit III (TEUF0084)	COIII	DQ343645
Cytochrome oxidase subunit III (TcTR-1480)	COIII	DQ343645
NADH dehydrogenase subunit 8 (CB923996)	*ND8*	DQ343645
Ribosomal protein S12 (TENF0522)	*RPS12*	AA676008

List of transcripts sequenced from IP-derived library used to identify putative UBP1 motifs. Excluding rRNAs and mitochondrial transcripts, the total number of clones analyzed was 40, including 32 sequences matching to CDSs and 8 to ESTs. The following cDNA clones were isolated 2 times: *ZFP1*, *PDH*, *AAT*, *CRUZ*, *HYPO3*, *TEUF0191*, and *TEUF0210*. The remaining sequences were sequenced once. *, not included in the dataset used for motif search.

**Table 2:** Experimental *Tc*RBP3-associated RNAs.

Description	Transcript Name	Systematic name or GenBank Acc. Num.
***mRNAs***		
Ubiquitin/Ribosomal protein S27a	*RPS27*	Tc00.1047053510409.39
Polypyrimidine-tract binding protein	*PTB*	Tc00.1047053511727.160
Ribosomal protein S2	*RPS2*	Tc00.1047053506213.60
Ribosomal protein P2	*RPP2*	Tc00.1047053510267.20
Ribosomal protein L5	*RPL18*	Tc00.1047053509671.80
Ribosomal protein S5	*RPS5*	Tc00.1047053506297.150
Ribosomal protein L22	*RPL22*	Tc00.1047053509747.20
Ribosomal protein L9*	*RPL6*	Tc00.1047053509695.170
Ribosomal protein L14*	*RPL14*	Tc00.1047053506861.30
Ribosomal protein S6*	*RPS6*	Tc00.1047053506241.170
Ribosomal protein L2*	*RPL2*	Tc00.1047053511181.100
Ribosomal protein S12*	*RPS12*	Tc00.1047053508231.20
Ribosomal protein L27a (L29)*	*RPL27*	Tc00.1047053508461.510
RNA helicase	*HELI*	Tc00.1047053511139.40
RHS family	*RHS*	Tc00.1047053508325.60
Sterol 24 C-methyltransferase	*CMT*	Tc00.1047053505683.10
Imidazolonepropionase	*IPROP*	Tc00.1047053508741.140
Flagellum-adhesion glycoprotein	*FAP*	Tc00.1047053503571.10
Simil S-adenosylmethionine decarboxylase	*SAM*	Tc00.1047053504257.30
Calpain-like cysteine peptidase	*CALP*	Tc00.1047053509001.40
Hypothetical protein 1	*HYPO1*	Tc00.1047053506811.120
Hypothetical protein 11	*HYPO11*	Tc00.1047053506949.30
Hypothetical protein 12	*HYPO12*	Tc00.1047053503999.80
		
***Expressed sequence tag (EST)***		
TENU 0711*	*EST7*	AI026499
		
***Ribosomal RNA***		
28S ribosomal RNA*	*28S *rRNA	L22334
		
***Mitochondrial transcripts***		
ATPase subunit 6*	*ATPase 6*	U43567
NADH dehydrogenase subunit 8 (TEUF0103)*	*ND8*	DQ343645
NADH dehydrogenase subunit 8 (TEUF0239)*	*ND8*	DQ343645

List of transcripts found in association with immunoprecipitated *Tc*RBP3. Excluding rRNAs and mitochondrial transcripts, the total number of clones analyzed was 25, including 24 sequences matching to CDSs and 1 to EST. *RPS2 *was isolated twice and the remaining sequences sequenced once. *, not included in the dataset used for motif search.

### Specific mRNA targets are differentially represented when comparing *Tc*UBP1- and *Tc*RBP3-containing mRNP complexes

**Figure 3:**
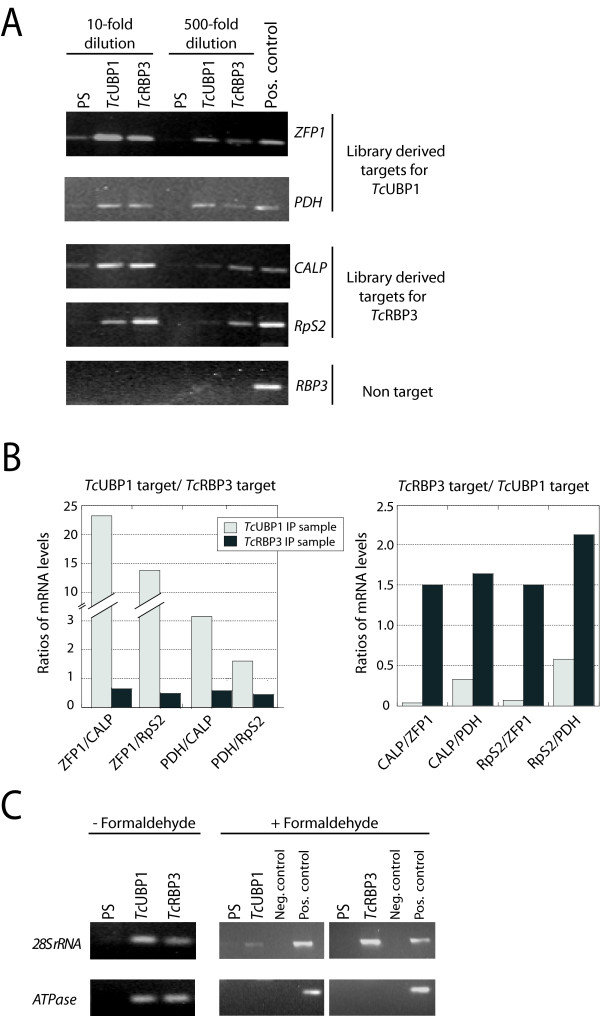
**Validation of experimental transcripts**. A) IP assays and semiquantitative RT-PCR amplification (25–30 cycles) were done to test library and non-related targets (*RBP3*) in the fraction of mRNAs bound to each protein. Specific set of primers shown at the right side of each panel were used. Only representative dilutions are shown. B) Abundance of transcripts present in *Tc*UBP1 and *Tc*RBP3 IP samples was assessed by real-time RT-PCR. Two mRNA targets for each protein in each IP sample were quantified. The relations of *Tc*UBP1 over *Tc*RBP3 targets in *Tc*UBP1, and in *Tc*RBP3 IP samples are plotted. The inverse relations are also shown. C) Parasites were *in vivo *treated with formaldehyde to maintain endogenous mRNP previous to IP and RT-PCR assays. PS, preimmune serum.

The presence of target mRNAs in mRNP complexes was validated in IP reactions using antibodies against individual RBPs. Bound RNAs were reverse transcribed and the presence of several randomly chosen targets from each library was analyzed by PCR. All transcripts expected to bind to *Tc*UBP1 or *Tc*RBP3 were confirmed to be present in each IP sample. A control mRNA encoding *Tc*RBP3 (*RBP3*), a transcript that does not bind to any of the RBPs tested, gave negative results. In all cases, samples were incubated with RNAse-free DNAse before reverse transcription and negative controls without reverse transcriptase were included to exclude the possibility of contamination with genomic DNA (results not shown). Non-specific RNA-protein associations were determined by parallel incubations with preimmune serum, and undetectable or low levels of PCR amplification signals were obtained from these controls (Fig. 3A). We and other authors [33,34] have observed PCR products in immunoprecipitates performed with control serum. However, these bands were in all cases much fainter than bands present in IP samples obtained using antibodies against the RBPs. Some *Tc*UBP1 targets derived from the library were found in *Tc*RBP3-IP material, and sequenced targets for *Tc*RBP3 were also present in the group of purified transcripts obtained using anti-*Tc*UBP1 antibodies. However, semiquantitative RT-PCR reactions demonstrated that specific transcripts were enriched in each sample (see the 500-fold dilution data in Fig. 3A). To further analyze these results, *Tc*UBP1- and *Tc*RBP3-associated transcripts in each IP were measured using real-time PCR. Specific primers for *Zinc Finger Protein 1 *(*ZFP1*) (Tc00.1047053511511.6), *Pyruvate dehydrogenase E1 subunit beta *(*PDH*) (Tc00.1047053510091.80), *Ribosomal protein S2 *(*RpS2*) (Tc00.1047053506213.60), and *Calpain-like cysteine peptidase *(*CALP*) (Tc00.1047053509001.40) were used. Real-time PCR was performed on samples immunoprecipitated with antibodies against *Tc*UBP1 or *Tc*RBP3, so it was not possible to use a reference transcript to standardize the assays. To overcome this drawback, we compared the ratios of the values obtained for each *Tc*UBP1 target mRNA over each *Tc*RBP3 transcript within *Tc*UBP1-IP or *Tc*RBP3-IP material (see Methods for details). The data presented in Figure 3B are representative of three independent assays. The results indicated that *Tc*UBP1 target transcripts were enriched in *Tc*UBP1 IP material. Thus, *ZFP1 *(a *Tc*UBP1 transcript target) levels were 24- and 14-fold more abundant than those of *CALP *and *RpS2 *(both *Tc*RBP3 targets), respectively; and *PDH *levels (*PDH *is also a *Tc*UBP1 transcript target) were 3- and 1.7-fold higher than those of *CALP *and *RpS2*. These ratios were lower in the group of *Tc*RBP3-associated transcripts (black bars in Fig. 3B, left panel).

Reciprocal analysis of the relative ratios of *Tc*RBP3 over *Tc*UBP1 targets in the pool of *Tc*RBP3-bound mRNAs showed that *CALP *levels were 1.5- and 1.6-fold higher than those of *ZFP1 *and *PDH*, respectively, and similar values (1.5 and 2.1, respectively) were found when *RpS2 *was analyzed. As anticipated, these ratios were significantly lower in the *Tc*UBP1-IP material (gray bars in Fig. 3B, right panel).

Mitochondrial transcripts and rRNAs were repeatedly found in both *Tc*UBP1- and *Tc*RBP3-IP samples. As these transcripts are highly abundant, and as neither RBP is localized in mitochondria ([35] and G. Noé, J. De Gaudenzi and A. C. Frasch, unpublished work), they probably correspond to background noise arising from non-physiological association during isolation [36]. To address this controversial result, and to obtain evidence for the occurrence of the observed interactions in intact cells, formaldehyde treatment prior to cell lysis was performed to crosslink RNP complexes in living parasites. Analysis of RNA present in IP-samples isolated from treated cells further supported the endogenous interaction of both proteins with rRNAs and confirmed several *Tc*UBP1- and *Tc*RBP3-associated transcripts as *in vivo *targets (Fig. 3C, and data not shown). In contrast, mitochondrial transcripts were not found in association with either RBP in cells treated by crosslinking, thus demonstrating that such interactions were non-physiological (Fig. 3C). In summary, these data allow us to conclude that most mRNAs identified in our libraries (Tables 1 and 2) are likely to be RBP-target transcripts. Although both *Tc*RBPs can share target transcripts, each preferentially binds a given set of mRNAs.

### Identification of conserved motifs for *Tc*UBP1 and *Tc*RBP3 binding in target transcripts

**Figure 4:**
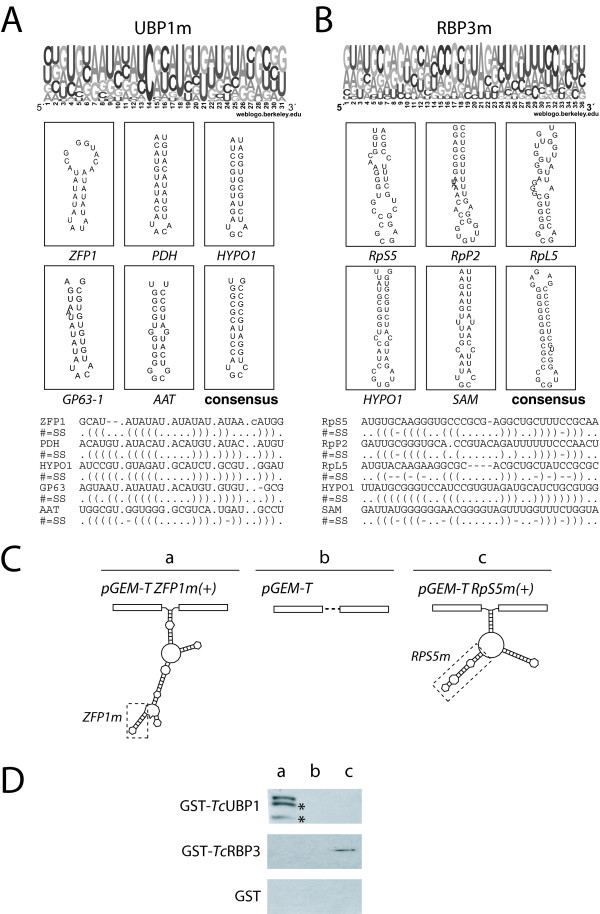
**Sequence, structure and validation of predicted motifs**. Probability matrix indicating the relative frequency of each residue at each position within the motif are shown, as well as secondary structures and structural alignment of five representative examples of motifs in specific transcripts. The alignment is represented by left and right balanced parenthesis indicating the pair of nucleotides that match by Watson-Crick complement or GU wobble pair. A) Best RNA motif identified for *Tc*UBP1 (UBP1m). B) Best RNA motif for *Tc*RBP3 (RBP3m). C) Scheme of the transcripts prepared for *in vitro *assays. PGEM-T was used as a control transcript because it lacks the motifs under analysis. Fragments of *ZFP1 *and *RpS5 *mRNAs bearing UBP1m and RBP3m, respectively, were inserted into PGEM-T. Dotted rectangle, location of motifs within each transcript. D) Biotin pull-down, GST, GST-*Tc*UBP1 and/or GST-*Tc*RBP3 (25 nM) were incubated with biotinylated transcripts (a) pGEM-T ZFP1m(+), (b) pGEM-T, (c) pGEM-T RpS5m(+). RNA-protein complexes were recovered using streptavidin-conjugated beads and detected by Western blotting. * degradation products of recombinant GST-*Tc*UBP1.

We next sought to determine whether each set of experimentally bound mRNAs might contain common sequence elements, using two motif-discovery tools (see Methods) based on both primary RNA sequences and secondary structures. Among several candidate motifs, one *cis*-element for each protein, termed UBP1m and RBP3m, were further analyzed. These were selected because they were present in most experimental targets identified but were of minor occurrence in the entire *T. cruzi *RefSeq database (see Table 3). A common stem-loop structure within these sequences was observed, suggesting that the *Tc*UBP1 and *Tc*RBP3 proteins might recognize structural elements rather than a particular sequence in the mRNAs. This means that different mRNAs might harbor the same structural motif even though they do not share the same sequence. Figure 4 shows the frequency of nucleotides at each position obtained from multiple alignment of the elements found within all of the target transcripts. Five representative examples of sequences and secondary structures in distinct mRNAs are also graphically represented (Figs. 4A and 4B).

**Table 3:** Number of hits (%) and frequency (hits/kb) of motifs in the experimental dataset and RefSeq database.

Motif	Parameter	Motif in dataset		Score Exp./RefSeq
			
		Exp. dataset	RefSeq	
UBP1m	%	70.27	9.48	-
	Frequency	1.75	0.11	16.52
RBP3m	%	70.59	5.21	-
	Frequency	1.09	0.06	19.39

RefSeq database is composed of trypanosome full length mRNAs and experimental dataset represent the 3' UTR of transcripts. The Score value represents the relationship between both frequencies.

Covariance model searches [37] allowed us to identify transcripts in databases that contained each of the motifs analyzed. These mRNAs are novel putative targets for each of the *Tc*UBP1 and *Tc*RBP3 proteins (database targets). A total of 1,547 additional EST targets (~10% of the total *T. cruzi *EST database) were identified using UBP1m in the search. In addition, 355 database transcripts (~2% of the database) bearing RBP3m were found. Table 3 shows the percentages of experimentally selected transcripts harboring the motifs and compares it with the percentages of transcripts in the RefSeq database (*T. cruzi-*filtered) bearing the motifs. The results show that the selected motifs are enriched in the experimental dataset compared with the entire transcriptome. Although most of the *Tc*UBP1 experimental targets (70.27%) contain one or two copies of UBP1m, some lack this motif, indicating that other motifs may also be relevant for *Tc*UBP1 binding. Similarly, RBP3m was represented in ~70% of the experimental dataset, which also suggests the existence of alternative motifs involved in *Tc*RBP3 binding (see following sections).

We next assigned relative frequencies to both motifs, reflecting the number of hits found in each group of transcripts divided by its sequence length (represented as hits per kb). UBP1m and RBP3m were 16.5- and ~19-fold, respectively, overrepresented in the experimental dataset compared with the *T. cruzi *RefSeq database (Table 3). Moreover, when the relative frequencies of motifs within 5'-upstream genomic sequences (5'-US), coding sequences (CDS), and 3'-downstream genomic sequences (3'-DS), of the experimental dataset and the *T. cruzi *CL Brener genomic sequence database were compared, a higher enrichment in the 3'-DS was observed, denoting a preferred 3'-UTR localization for both motifs (Table 4). Taken together, these results demonstrate that the structural elements identified in trypanosomal transcripts are conserved and enriched within the experimental set of transcripts and that these motifs are preferentially localized in the 3'-UTRs.

**Table 4:** Relative frequency of motifs in 5' US, CDS and 3' DS in experimental and TcruziDB databases.

Motif	Molecule region	Experimental dataset	TcruziDB
	5' US	0.00	0.35
UBP1m	CDS	0.10	0.08
	3' DS	2.07	0.81

	5' US	0.00	0.04
RBP3m	CDS	0.17	0.06
	3' DS	0.72	0.16

Frequency (hits/kb) of motifs in experimental and TcruziDB CL Brener genomic sequence Release 5.1 fractioned in 3 subsets compose of 5' US, CDS and 3'DS (see Methods for details).

### RNA motifs enhance RNA-*Tc*UBP1 or -*Tc*RBP3 interactions

The capacity of each RRM-type protein to bind biotinylated transcripts harboring the described motifs was tested. The RNA-protein complexes were pulled-down with streptavidin-coated beads and the presence of the protein revealed by Western blotting. ZFP1m is a short region of the *ZFP1 *mRNA that contains UBP1m, whereas RpS5m is a portion of the *RpS5 *transcript harboring RBP3m (Fig. 4C). Both fragments were inserted into a pGEM-T polylinker transcript and are termed pGEM-T ZFP1m(+) and pGEM-T RpS5m(+), respectively. A pGEM-T polylinker transcript without any insert was used as a negative control because *in silico *predictions indicated that this sequence did not contain any of the motifs under analysis. RNAs were transcribed *in vitro *in the presence of CTP-Biotin and incubated with recombinant GST-tagged *Tc*UBP1, *Tc*RBP3, or GST alone (as a control). pGEM-T ZFP1m(+) effectively pulled-down *Tc*UBP1 protein but failed to show binding to *Tc*RBP3 or to control GST protein. In contrast, pGEM-T RpS5m(+), pulled-down *Tc*RBP3 but not *Tc*UBP1 or GST (Fig. 4D). The control transcript (pGEM-T) failed to show binding to any of the *Tc*RBPs.

**Figure 5:**
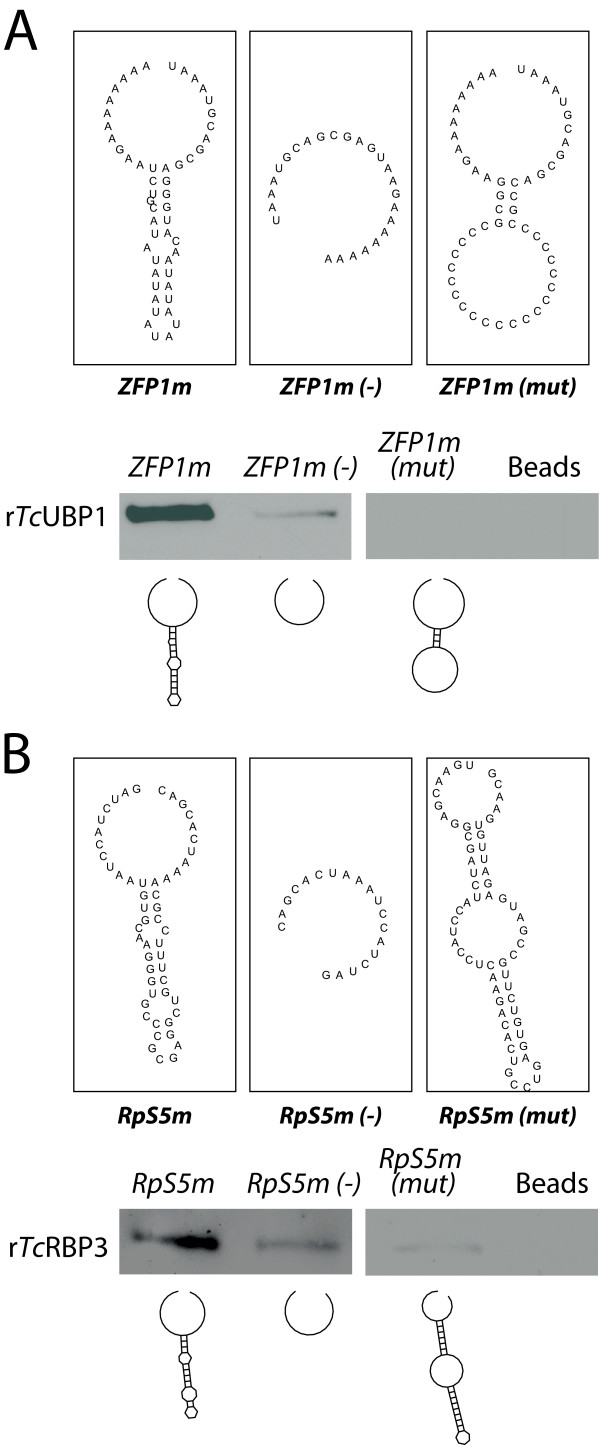
**Deletions and mutations of motifs sequences hinder the interaction with proteins**. Biotin pull-down assays using shorter and mutated regions of *ZFP1 *and *RpS5 *mRNAs. Secondary structures of these RNAs are shown. ZFP1m and RPS5m are RNAs comprising flanking regions plus UBP1m or RBP3m, respectively. ZFP1m(-) and RPS5m(-) contain only flanking regions and ZFP1m (mut) and RPS5m (mut) enclose irrelevant motifs. GST fusion proteins (r*Tc*UBP1 and r*Tc*RBP3) were recognized by Western blot.

To analyze if the stem-loop structures identified might be involved in the interaction of proteins with target mRNAs, the following transcripts were transcribed and used in RNA-binding assays: (1) RNAs comprising short flanking regions with either UBP1m or RBP3m (ZFP1m and RPS5m); (2) RNAs lacking the motifs but containing only the flanking regions [ZFP1m(-) and RPS5m(-)]; and, (3) RNAs in which the motifs of interest were replaced by different sequences [ZFP1m (mut) and RPS5m (mut)]. As shown in Figure 5A and 5B, weak binding was seen when the motifs were absent [ZFP1m(-) and RPS5m(-)] or when they were substituted by other sequences [ZFP1m (mut) or RPS5m (mut)]. It is to be noted that RBP3 did not bind to RpS5m (mut) construct albeit it folds into a stem-loop structure. Besides, it did not bind other predicted stem-loops such as the one present in the *ZFP1 *3'-UTR showed in Figure 4D, indicating that slight differences in the RNA structure affect RNA-binding. Contrary, transcripts bearing complete UBP1m or RBP3m motifs showed considerably enhanced interactions with the corresponding RBPs. Taken together, these results demonstrate that the identified RNA elements are involved in interactions with RBPs and that transcripts can be rendered targets for UBP1 or RBP3 by addition of the correct RNA motif.

### Assessment of the validity of novel target transcripts identified in databases

**Figure 6:**
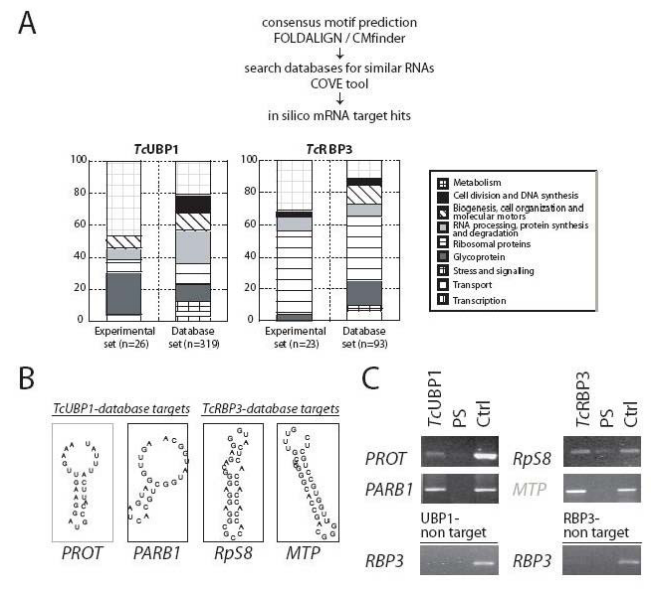
**Classification of mRNAs interacting with proteins and validation of database targets**. A) New and potential targets were found by searching databases for mRNA harbouring UBP1m or RBP3m. Column charts show a functional classification of gene products encoded by experimental and potential targets. B) Secondary structures of representative examples of motifs and validation of database targets by RT-PCR in the IP material. *PROT*, 29 KDa Proteasome subunit TCPR29; *PARB1*, Paraflagellar rod component 1; *RpS8*, Ribosomal protein S8; MTP, Membrane transporter protein.

To test the usefulness of UBP1m and RBP3m elements in predicting novel targets, we first manually classified (into nine functional categories) all motif-containing sequences obtained from dbEST, which had been previously filtered using the annotation file provided by TIGR [38] (Additional files 2 and 3). Target mRNAs present in each experimental dataset were also classified (as listed in Tables 1 and 2). The overall distribution of transcripts in these groups was similar when comparing database and experimental targets for each of *Tc*UBP1 and *Tc*RBP3 proteins, but was different when the comparison was performed between targets of both proteins (Fig. 6A). Thus, a statistically significant difference (chi-square, p < 0.001) was found between the distribution of UBP1 and RBP3 database targets. Although mRNAs from various categories were identified, groups with larger numbers of transcripts were found. mRNAs coding for proteins involved in general metabolic pathways are overrepresented in both datasets of *Tc*UBP1 targets analyzed. Conversely, RPC transcripts are the most distinctive group among *Tc*RBP3 database and experimental targets.

We then investigated whether some of the putative database targets identified *in silico *might indeed be able to interact with the corresponding RBP. *In vivo *IP assays were performed and RT-PCR was used to detect transcripts encoding the *29-kDa proteasome subunit class II *mRNA (*PROT*) (TCU75302) and *Paraflagellar rod component 1 *mRNA (*PARB1*) (Tc00.1047053506755.20), both of which are putative targets for *Tc*UBP1, and *Ribosomal protein S8 *mRNA (*RpS8*) (Tc00.1047053511903.110) and *Membrane transporter protein *mRNA (*MTP*) (Tc00.1047053511307.3), both of which are predicted targets for *Tc*RBP3. The association of database targets with the two proteins was then investigated by RNA extraction from purified mRNP complexes followed by RT-PCR. All database targets studied were found in the pool of bound RNAs (Fig. 6B). In summary, the motifs identified for *Tc*UBP1 or *Tc*RBP3 binding can be effectively used to identify novel target transcripts for both RBPs. Furthermore, these results allowed us to recognize groups of mRNAs with common functions as potential binding partners for each protein.

### Common target RNAs for *Tc*UBP1 and *Tc*RBP3

**Figure 7:**
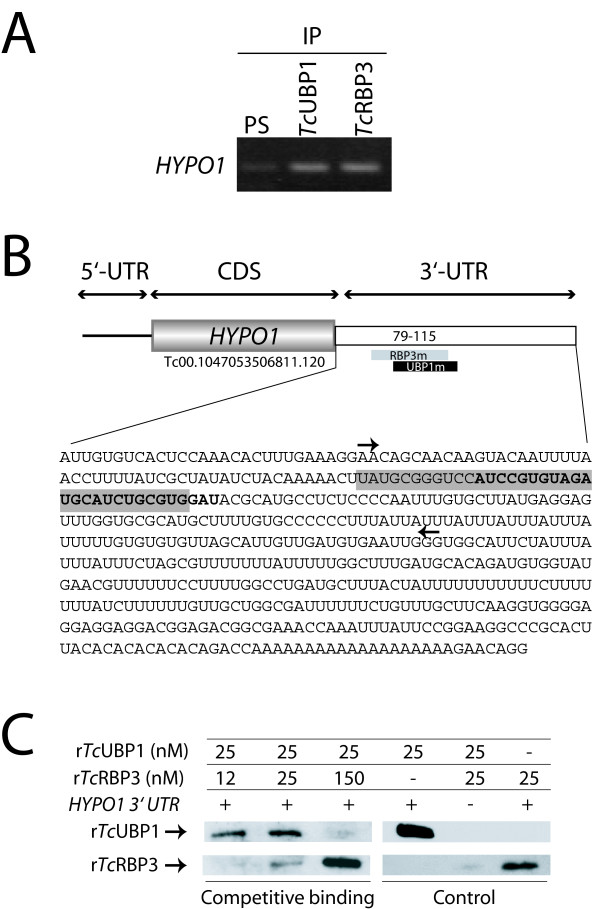
***In vitro *binding of *Tc*UBP1 and *Tc*RBP3 to a common target**. A) *HYPO1 *was identified in both libraries. The interaction with each protein was confirmed by RT-PCR assays in IP samples. B) Scheme of *HYPO1 *mRNA, 3' UTR sequence and location of UBP1m (black or bold) and RBP3m (gray) are shown. *In vitro *transcribed region is marked with arrows. The numbers indicate distances from stop codon where motifs were found. C) *In vitro *binding reactions were performed incubating the biotinylated transcript with both recombinant proteins in the same binding reaction at the concentrations indicated.

Based on the above data on RNA-protein interactions, demonstrating that both RBPs can share transcripts, we sought to identify whether some targets might also contain both previously described motifs, UBP1m and RBP3m. Eleven database transcripts might potentially be common to both proteins (Table 5). This list includes RPC genes (*RpS6, RpL29*), *PDH*, and *Histones*, among others. Interestingly, the *HYPO1 *gene (Tc00.1047053506811.120) is a library-derived target for both RBPs (Fig. 7A, Tables 1 and 2), bearing a region in which UBP1m intersects RBP3m. Here, the two motifs are superimposed in the same region inside the 3'-UTR (RBP3m nt 79–112, UBP1m nt 90–115) (see Fig. 7B). *In vitro *binding assays were performed to investigate whether both proteins could bind to and compete for this region. As shown in Figure 7C, increasing amounts of *Tc*RBP3 in the presence of fixed amounts of *Tc*UBP1 caused a reduction in binding to *Tc*UBP1. These observations showed that both proteins bind to common sites in *HYPO1 *3'-UTR in a competitive manner and support the view that *Tc*UBP1 and *Tc*RBP3 can share RNA targets.

**Table 5:** Common mRNA targets bearing the *cis*-elements UBP1m and RBP3m included in experimental and database lists.

*Systematic name or TIGR identifier*	*Description*
Tc00.1047053506811.120	Hypothetical protein 1 *(HYPO1)*
UP|Q9NIQ8 (Q9NIQ8)	Mucin-like protein
UP|Q72DX6 (Q72DX6)	Sensor histidine kinase
UP|Q25325 (Q25325)	Heat shock protein 70-related protein
UP|Q7TPK7 (Q7TPK7)	Ac2-048
UP|Q9XY95 (Q9XY95)	Neurotrophin
UP|H2A_TRYCR (P35066)	Histone H2A
UP|RL29_DROME (Q24154)	60S ribosomal protein L29 (L43)
UP|RS6_LEIMA (Q9NE83)	40S ribosomal protein S6
PDB|1II2_A.0|17942708|	1II2_A Chain A, Crystal Structure Of Phosphoenolpyruvate Carboxykinase (Pepck)
UP|O48239 (O48239)	Cytochrome b (Fragment)
gb|L22334.1|TRBS3RRBN	5.8S ribosomal RNA, internal transcribed spacers 1–7, and 28S ribosomal RNA

### Additional motifs can also govern the binding of *Tc*RBPs to target transcripts

**Figure 8:**
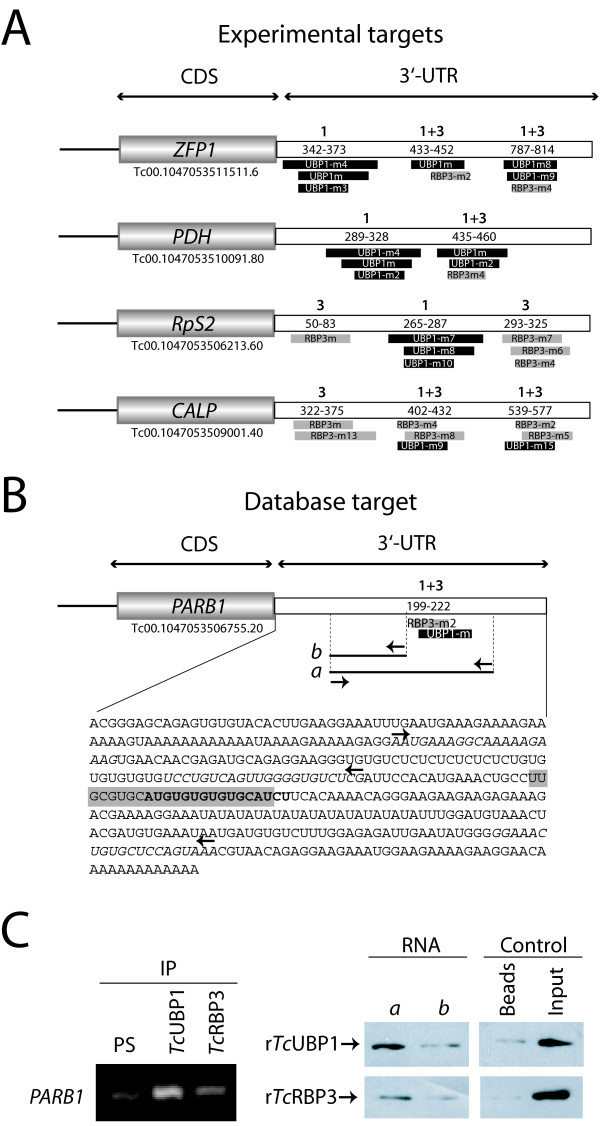
***Tc*UBP1 and *Tc*RBP3 bind to a transcript containing alternative RNA motifs**. A) Scheme showing the spatial distribution of additional motifs in 3' UTR of specific transcripts. 1 and 3, represent a region where different motifs exist for *Tc*UBP1 and *Tc*RBP3, respectively; 1+3, represent regions where overlapping motifs for both proteins were found. Lines below them represent a motif. B) Scheme of *PARB1 *mRNA and its 3' UTR containing additional motifs for each protein (named UBP1-m14 and RBP3-m2). The numbers indicate distances from stop codon where motifs were found. C) RT-PCR assays in IP samples shows that *PARB1 *is a common target. Biotin pull-down assay was used to asses the binding of both proteins to the following regions of *PARB1 *mRNA: a) with both motifs b) without motifs.

To obtain additional evidence for the existence of common transcript targets for both RBPs, we analyzed the list of other possible *cis*-elements mentioned above. About 15 motifs enriched in the group of experimental targets for each RBP were found (Table 6), and all shared a common stem-loop structure. The overall distribution of motifs within some transcripts was analyzed. A 'hot spot' region for motifs was observed, and some overlapped at the primary sequence level, suggesting that binding sites could not be defined simply by structure. *Tc*RBP3 motifs can be found in some *Tc*UBP1-bound mRNAs and *vice versa*. There are also regions bearing *Tc*UBP1 and *Tc*RBP3 motifs. For example, a portion of UBP1m in the *ZFP1 *3'-UTR coincides with UBP1-m3 and UBP1-m4. Downstream, the putative motifs detected for *Tc*UBP1 extend beyond the potential motif for *Tc*RBP3 (see Fig. 8A).

**Table 6:** List of all candidate motifs found for both RBPs, *Tc*UBP1 and *Tc*RBP3.

	% of mRNAs		
		
RNA motifs	EXP	dbEST	Δ
UBP1m	70.27	9.74	61
UBP1-m2	56.76	20.26	36
UBP1-m3	51.35	15.52	36
UBP1-m4	35.14	1.22	34
UBP1-m5	37.84	5.32	33
UBP1-m6	43.24	12.06	31
UBP1-m7	43.24	17.39	26
UBP1-m8	51.35	25.66	26
UBP1-m9	43.24	17.78	25
UBP1-m10	37.84	14.96	23
UBP1-m11	32.43	10.17	22
UBP1-m12	24.32	3.39	21
UBP1-m13	27.03	6.60	20
UBP1-m14	27.03	8.18	19
UBP1-m15	27.03	8.52	19
UBP1-m16	8.11	0.31	8

RBP3m	70.59	2.23	68
RBP3-m2	70.59	8.69	62
RBP3-m3	88.24	29.09	59
RBP3-m4	82.35	26.02	56
RBP3-m5	58.82	6.83	52
RBP3-m6	47.06	5.76	41
RBP3-m7	41.18	2.81	38
RBP3-m8	41.18	4.05	37
RBP3-m9	35.29	2.61	33
RBP3-m10	29.41	0.13	29
RBP3-m11	29.41	1.20	28
RBP3-m12	23.53	0.07	23
RBP3-m13	17.65	0.04	18
RBP3-m14	17.65	0.48	17

Percentage of transcripts bearing the motif in the experimental dataset and trypanosome EST database are shown. Δ, indicates the difference between both percentages.

Some mRNAs contain several motifs for either *Tc*UBP1 or *Tc*RBP3, indicating that certain RNA signals may be redundantly present. Thus, binding of both RBPs might occur at distinct sites of 3'-UTR target mRNAs (Additional file 4). In accordance with previous results, *Tc*RBP3 database targets with several motifs include RPC genes [*RpS10 *(Acc. Num. AA926483), *RpL29 *(Acc. Num. AA835614), and *RpL38 *(Acc. Num. CF888343)] were found. To determine if the additional motifs identified might be functional in terms of binding to transcripts, a region of *PARB1 *3'-UTR that contains overlapping motifs (UBP1-m14 and RBP3-m2) for each protein was analyzed (Fig. 8B). First, RT-PCR with IP samples was performed to show that *PARB1 *3'-UTR is a target for both proteins. Second, the ability of both RBPs to bind the region of the *PARB1 *3'-UTR that contains UBP1-m14 and RBP3-m2 was confirmed (a in Fig. 8C). In contrast, both RBPs were incapable of interacting with a portion of the UTR deficient in these motifs (b in Fig. 8C). These data demonstrate that *Tc*RBPs can bind to more than one motif and suggest that subsets of cellular targets for each protein may be determined by the combination of different possible motifs within these transcripts.

## Discussion

The functionality of the RNA molecule depends not only on primary sequence information but also on secondary folding. Work on RNA motif discovery is relevant to better understand the metabolism of RNA, which essentially relies on interaction of transcripts with proteins. In recent years, high-throughput techniques have been developed to map post-transcriptional networks; these combine the isolation and identification of mRNAs from cell extracts (such as the RNP IP-microarray/Chip [39]) with Ribotrap analysis to determine bound proteins [40]. Methods to detect structural elements in a set of RNA molecules were recently published [41], and a web server for RNA data analysis, with significant capabilities for RNA secondary structures prediction was released [42]. In this work, RNA structural elements were identified in target transcripts of two different RRM-containing trypanosomal RBPs [25,26,29,35]. This subject is particularly relevant to work with Kinetoplastid parasites, because gene expression in such cells is basically controlled at the post-transcriptional level. It is known that regulatory regions of transcripts contain *cis*-elements that determine the fate of monocistronic mRNAs by interaction with specific *trans*-acting RBPs, but much work remains to be done to determine these molecular mechanisms [43]. Targets for trypanosome RRM-type proteins have been studied for only a few RBPs, such as the PUF family members in *T. brucei *and *T. cruzi *[44-46]. In the present work, a library of transcripts extracted from *Tc*UBP1- or *Tc*RBP3-containing mRNP complexes allowed the identification of target mRNAs. Signature motifs in these transcripts were found and shown to be useful, in turn, in the prediction of other putative transcript targets in databases. Some of these mRNAs were shown to be complexed with the relevant proteins, thus confirming the feasibility of the approach for the identification of groups of transcripts binding to a given RBP.

Distinct categories of sequence motifs involved in multiple levels of mRNA metabolism have previously been mapped in trypanosomes (reviewed in references [19] and [47]). For example, a 26-mer U-rich bloodstream-form instability element [48] and a 16-mer stem-loop required for efficient translation [49] were both described in the 3'-UTR of the *EP procyclin T. brucei *transcript. These regulatory signals are common to several genes. In *Leishmania*, a 450-nt 3'-UTR element conserved among a large number of *amastin *mRNAs is responsible for stage-specific gene regulation [50], and a consensus motif UAUUUUUU has also been described for the nuclear-encoded components of the cytochrome oxidase complex in *T. brucei *[51]. The *cis*-elements UBP1m and RBP3m characterized in this work are the best signature motifs for the RBPs examined here, and they are necessary for RNA-binding activities. However, the motifs were present in only a subset of experimental transcripts, indicating that several sequence elements might be involved in RNA-protein recognition. Therefore, our findings suggest the existence of a wide variety of RNA target motifs for each factor, as was previously described for other RBPs such as TIA-1, TIAR, HuR, and Puf proteins [33,34,52]. Additional motifs were also functional in *Tc*RBP binding and we found 3'-UTRs in several transcripts bearing different motifs for either *Tc*UBP1 or *Tc*RBP3 (Additional file 4). Bibliographical reports indicate that redundancy of *cis*-elements constitutes a mechanism to ensure or modulate the functionality of RNA-protein interaction with a given target transcript [53,54].

Motifs conserved within the group of *Tc*RBP3-bound transcripts were also found in a number of *Tc*UBP1 experimental mRNA targets, and *vice versa*, and some of these regions overlapped. For instance, *HYPO1 *mRNA, a library-derived target for both *Tc*RBPs, had a 3'-UTR where UBP1m intersected RBP3m. This region was able to bind both proteins, similar to what was recently described for binding of the yeast Puf1p and Puf5p family members to a motif within the *TIF1 *3'-UTR [54]. More generally, we have demonstrated here that both proteins might share and bind to common targets, as has been published for HuR and AUF1 proteins that bind to many common AU-rich transcripts [55,56], and yeast Puf4p and MpT5p that negatively regulate a single mRNA [57].

Results shown in Figure 3 suggest that experimental targets for each protein, *Tc*UBP1 or *Tc*RBP3, could also be associated with the other protein. Although these findings are consistent with the relatively non-specific binding capacity of *Tb*UBP1 and *Tb*UBP2 postulated in *T. brucei *[29], real-time PCR experiments have provided evidence that each RBP preferentially binds a given group of transcripts (Fig. 3B). Thus, the association of mRNAs with *Tc*UBP1 or *Tc*RBP3 probably depends on many dynamic factors, including: (1) the abundance of the transcript itself; (2) the combination of multiple elements the transcript bears in the 3'-UTR; (3) the affinity of each protein for the different motifs; (4) the accessibility of each RBP and additional mRNA binding factors; and, (5) the condition of the parasite at any given moment. In this regard, it has recently been reported that during nutritional stress *Tc*UBP1 and *Tc*RBP3 re-localize in large cytoplasmic granules [35] containing other RRM-type proteins and polyadenylated mRNAs. Interestingly, under normal conditions RBPs are also present in discrete, but small, cytoplasmic granules (G. Noé, J. De Gaudenzi and A. C. Frasch, unpublished data). The composition of these mRNP granules might determine the fate of the transcripts they contain under natural conditions. Further work should indicate the minimal set of RBPs associated with a single transcript and will measure the stability, translation, or degradation of such transcripts.

RNA-binding domains are well conserved among Kinetoplastids, so the presence of the identified RNA motifs in ortholog mRNA targets from *T. brucei *and *L. major *was investigated. To this end, we obtained trypanosomatid transcripts that were orthologs of the experimental targets using the GeneDB web server , and searched for RNA structural motifs. Interestingly, more than 80% (27 of 32) of the UBP1 ortholog transcripts contained UBP1m and 32.4% (12 of 37) of the ortholog RBP3 targets encompassed RBP3m (data not shown), suggesting that these RRM proteins could share a similar set of mRNA targets in the three trypanosomatids. Functionally related groups of transcripts within the list of experimental and database targets for each protein were found. The model that emerges from our results is in line with the results of an increasing number of studies [58-60] that suggest coordination of gene expression by combinatorial binding of RBPs to different subsets of functionally related mRNAs, thus defining a post-transcriptional operon that increases regulatory flexibility following biological perturbations. Many of the mRNAs identified in *Tc*UBP1-containing mRNP complexes encode proteins involved in general metabolic pathways. Additionally, we found mRNAs encoding several surface proteins, including *SMUG *mRNA, a known target for *Tc*UBP1 [26]. Notably, one of the sequences found matches the 3'-UTR of four glycoprotein-coding genes (*Gp85*, *Mucin-like protein*, *Host cell signaling surface protein *and *Trypomastigote surface antigen TSA-1*); all possess nearly the same sequence in their 3'-UTRs and harbor UBP1m (data not shown). Among the *Tc*RBP3-associated transcripts experimentally obtained, and also confirmed in databases (Fig. 6), a large number of RPC genes were found, thus suggesting the possibility that *Tc*RBP3 may regulate and coordinate ribosome biogenesis, as has been previously described for other RRM-type proteins [61,62]. As mentioned, the observations are consistent with the idea that functionally related mRNAs of trypanosomes might be coordinately regulated at the post-transcriptional level by specific RBPs, in agreement with the RNA operon/regulon model suggested for other cells (reviewed in reference [60]). This or other alternatives are interesting in the context of a single cell, the trypanosome, which likely makes RNA metabolism the sole mechanism to regulate gene expression.

## Conclusion

In this paper we identified and validated target mRNAs for two phylogenetically conserved RRM-containing RBPs among Kinetoplastids. *Tc*UBP1 and *Tc*RBP3 can share target transcripts, although they preferentially bind given sets of mRNAs. These trypanosomal target transcripts contain conserved structural elements, involved in RNA-binding, in their 3'-UTRs. Moreover, the elements identified for *Tc*UBP1 or *Tc*RBP3 binding were successfully used to determine novel database targets that were classified within groups of common functions. In addition, we found that trypanosome RBPs can associate with more than one motif, and the combination of elements is the main factor in determination of RNA-protein recognition.

## Methods

### Parasite cultures and transfections

*T. cruzi *CL Brener cloned stock was used [63]. Culture conditions and protein extract preparations were according to Di Noia *et al*. [64].

### Databases

Trypanosome database (*T. cruzi *CL Brener genomic sequence Release 5.1) utilized in this work was obtained from TcruziDB server . 5' upstream genomic sequences (5' US) and 3' downstream genomic sequences (3' DS) were obtained using TcruziDB sequence retrieval tool. A length of 50 nt upstream to the CDS was used to obtain sequences resembling the 5' UTR, while 300 nt downstream to CDS were used for 3' UTR, in agreement to previously reported data from trypanosomes [65]. Reference Sequence (RefSeq) and EST databases (*T. cruzi *filtered) were downloaded from NCBI. The TIGR *T. cruzi *Gene Index database  was used to analyze and classify all *T. cruzi *EST sequences.

### Western blot analysis

Protein samples resolved by SDS-PAGE gels were transferred onto Hybond C nitrocellulose membrane (Amersham Pharmacia Biosciences), probed with primary antibodies and developed using horseradish peroxide conjugated anti rabbit antibodies and Supersignal^® ^West Pico Chemiluminescent Substrate (Pierce Biotechnology). The antibodies used in this work were: polyclonal rabbit anti-peptide antibody reacting with amino-terminal domain of *Tc*UBP1 (anti-*Tc*UBP1) and anti-*Tc*RBP3 serum raised against the complete protein (anti-*Tc*RBP3) [26].

### Immunoprecipitation (IP) assays

A cytosolic protein extract corresponding to 10^9 ^parasites was precleared for 30 min at 4°C using rabbit preimmune serum and 50 μl of protein A-Sepharose beads (Sigma) that had been previously swollen in Tris-buffered saline (TBS). Extract was then incubated with preimmune serum (as a control), a non-RBP serum anti-*T. cruzi *Ribose 5-phosphate isomerase Type B (*Tc*Rpi, used as a control) [66], anti-*Tc*UBP1 or anti-*Tc*RBP3 serum at 4°C with gentle mixing during 16 h. Beads (100 μl) were added to the mixture and after 2 h, extensive washes were made with TBS supplemented with 0.2% Tween-20. An aliquot (10% of the sample) was separated to asses the present of proteins in the immunoprecipitated material, proteins were extracted using Laemmli buffer and detected by Western blotting. The rest of the sample was used for RNA extraction using TRIzol reagent (Invitrogen) following manufacturer's instructions.

### *In vivo *formaldehyde fixation of parasites

Formaldehyde was added to *T. cruzi *cell cultures to a final concentration of 1% (v/v) and incubated at 28°C for 15 min with mixing. Crosslinking reactions were quenched by addition of glycine (final concentration, 250 mM) and incubation at RT for 5 min. Cells were washed twice with ice-cold PBS, resuspended in lysis buffer (20 mM Tris-HCl pH 7.6, 2 mM MgCl_2_, 10% glycerol, 0.5% NP-40, 1 mM EDTA, 1 mM DTT, 0.25 M sucrose and 50 mM KCl) and lysed by 3 rounds of sonication, 30 s each in a Branson 450 sonifier with 0.25 vol of 400 microns glass beads (Sigma). The lysate was centrifuge at 11 000 g for 10 min at 4°C and the supernatant was used for IP assays. Reversion of crosslinkings was done by incubation of protein-A-Sepharose beads in 50 mM Tris-HCL pH 7.6, 5 mM EDTA, 10 mM DTT and 1% SDS during 45 min at 70°C. RNA was extracted from IP material using Trizol and the presence of proteins confirmed by Western blot analysis.

### Library constructions and RT-PCRs

RNA extracted from the mRNP complexes was used to perform reverse transcription-polymerase chain reaction (RT-PCR) and to construct the library using BD Smart™ PCR cDNA synthesis Kit (BD Biosciences) according to manufacturer's instructions. PCR products were cloned into pGEM-T Easy vector (Promega) and sequenced. RT-PCRs of RNA extracted from mRNP complexes and serial dilutions of the samples were also used to detect the presence of target mRNAs using gene specific oligonucleotides (Additional file 5).

### Computational analysis

Modifications to the method previously described by Silanes *et al*. were done [33]. The following protocol was performed for both sets of co-immunoprecipitated sequences (*Tc*UBP1 and *Tc*RBP3 sets). Briefly, trypanosome records were identified by doing BLAST against five different databases (TcBr v2.0 genomic DNA, TcruziDB v5.1 CDS, Swissprot, NR, NT and RefSeq). More than 20 target transcripts (including 3' UTRs) for each gene were entered in two motif prediction tools using default motif range (30–100 bp). Using FOLDALIGN [67], sequences up to 550-bases were divided into 100-bases long subsequences with 50-bases overlap between consecutives sequences and were organized into 8 random data sets. Consensus motifs were predicted from each data set. Using CMfinder [68], sequences up to 500-base long were submitted to the web server . Candidate motifs obtained with both programs were used to build the stochastic context-free grammar (SCFG) model (COVE program). The SCFG for each candidate motif was used to search against the experimental 3' UTR data set and the RefSeq database to obtain the number of hits for each motif (COVELS program) [37]. The motif with the highest enrichment in the experimental data set over the literature database was considered to be the best candidate motif. The motif logo was constructed using WebLogo . Finally, RNAfold tool [69] was used to plot the secondary structure of the representative RNA motifs. The alignments were done using the on-line workbench server from the University of California, San Diego .

### Plasmid constructions

Regions from 3' UTRs of motif-containing transcripts were amplified by PCR using specific primers (Additional file 6) and cloned into pGEM-T Easy vector (Promega). The following genes (with the amplified fragments indicated between parentheses) were used in this work: *ZFP1 *(372 to 425), *RpS5 *(109 to 207), *HYPO1 *(29 to 158) and *PARB1 *[184 to 280 (a) and 184 to 464 (b)]. Construction of mutated/deleted stem-loop sequences was done by annealing of synthetic complementary oligonucleotides (see Additional file 6) followed by ligation into pBS(-) (Stratagene). Oligonucleotides were mixed, heated to 95°C and allowed to cool slowly producing double-stranded oligonucleotides with added *Eco*RI and *Hind*III cohesive ends in 5' and 3' ends, respectively, that were inserted by ligation into pBS vector previously digested with those enzymes.

### *In vitro *Biotin pull-down assay

pGEM-T Easy and pBS(-) plasmids containing the motifs were digested with *SpeI *and *Hind*III, respectively, for *in vitro *transcription with T7 RNA polymerase (Promega) and biotin-14-CTP (Invitrogen). The integrity of each biotinylated RNA was verified in 1.5% agarose gels. Purified recombinant GST-*Tc*UBP1, GST-*Tc*RBP3 or GST alone (25 nM) were incubated with 10-fold molar excess of biotin-RNA in Binding buffer (BB) (100 mM KCl, 1 mM DTT, 1% glycerol, 1 mM Cl_2_Mg, 20 mM Tris-HCl pH 7.6, 400 μg/ml tRNA, 200 μg/ml BSA and 10 units of RNase inhibitor) in a final volume of 50 μl for 30 min at room temperature (RT). 20 μl of Streptavidin-paramagnetic beads (Promega) previously washed in BB were added and incubated for 10 min at RT. Magnetic beads were then isolated and washed 5 times with BB supplemented with 1% Tween-20 and boiled in 2× Laemmli buffer to detect proteins by Western blotting. For competitive binding assays, increasing levels of *Tc*RBP3 (12.5, 25 and 150 nM) were incubated with a fixed concentration of *Tc*UBP1 (25 nM).

### Real-time PCR analysis

For RT-PCRs, 50% of total RNA isolated from IPs was reverse transcribed using oligo(dT) primer and SSII RT (Invitrogen). Products were diluted in water and amplified by quantitative PCR using specific primers (Additional file 5) in a Gene Amp 5700 Sequence detection system (Perkin Elmer, Applied Biosystem). PCR was carried out in a final volume of 12.5 μl reaction mixture containing 0.3 μM of each primer, 1× SYBR Green reaction mix (Applied Biosystem) and 4 μl of cDNA template. Standard curves were prepared for each run using known quantities of DNA and estimations of DNA levels were obtained using the Sequence detection System data analysis software. Two experimental targets were measured for *Tc*UBP1 (*ZFP1 *and *PDH*) and two transcripts for *Tc*RBP3 (*CALP *and *RpS2*) in *Tc*UBP1- and in *Tc*RBP3-IP materials. Relations of quantities found for *Tc*UBP1 over *Tc*RBP3 targets were done for each IP, as well as the inverse relation (*Tc*RBP3 over *Tc*UBP1 transcripts). The data was analyzed by comparing those relations in each IP.

## Abbreviations

ARE: AU-rich element; GST: glutathione S-transferase; IP: immunoprecipitation; mRNP: messenger ribonucleoprotein; UTR: untranslated region; RBP: RNA-binding protein; RRM: RNA-recognition motif; *Tc*UBP1: *T. cruzi *U-rich RNA-binding protein 1; *Tc*RBP3: *T. cruzi *RNA-binding protein 3.

## Authors' contributions

GN, JGDG and ACF conceived and designed the study. GN and JGDG carried out all experiments. ACF coordinated and supervised the work. All authors participated in writing of the manuscript and approved the final version.

## Supplementary Material

Additional file 1 **Immunoprecipitation control and RT-PCR.** As an internal control, IP and RT-PCR reactions were performed using a non-related antibody, anti-*Tc*Rpi. The same conditions and amplification cycles were used. A) Western blot employing anti-*Tc*Rpi in immunoprecipitated samples. The position of the bands corresponding to each protein and to the heavy chain (hc) of antibodies is indicated with an arrow on the left side of the panel. Molecular mass protein standards Dalton Mark VII-L™ are indicated on the right side. B) PCR amplification using specific sets of primers to confirm the absence of some identified transcripts for *Tc*UBP1 and *Tc*RBP3. PS, preimmune serum; *Tc*Rpi, *T. cruzi *Ribose 5-phosphate isomerase Type B. Click here for file

Additional file 2 **List of database targets harboring UBP1m.** Sequences obtained from dbEST bearing UBP1m were filtered using the annotation file provided by TIGR and manually classified into functional categories. N, number of sequences found. Click here for file

Additional file 3 **List of database targets bearing RBP3m.** Sequences obtained from dbEST bearing RBP3m were filtered using the annotation file provided by TIGR and manually classified into functional categories. N, number of sequences found. Click here for file

Additional file 4 **Several transcripts have different motifs for either *Tc*UBP1 or *Tc*RBP3.** This table contains a list of mRNA target hits bearing the highest four UBP1- and RBP3-motifs. Click here for file

Additional file 5 **PCR primers.** This table contains a list of oligonucleotides used in this work for RT-PCR. Click here for file

Additional file 6 **Primers for motifs and deletions.** This table contains a list of oligonucleotides used in this work for construction of motifs and deletions. Click here for file
