# Supplementary figures and images for: Functionally related transcripts have common RNA motifs for specific RNA-binding proteins in trypanosomes
Source: BMC Mol Biol. 2008 Dec 8;9:107. doi: 10.1186/1471-2199-9-107 (PMC2637893; doi:10.1186/1471-2199-9-107)

A

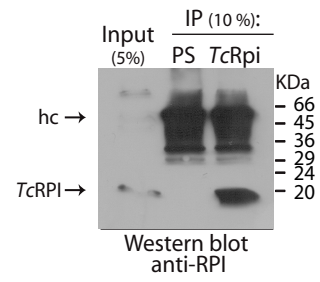

B

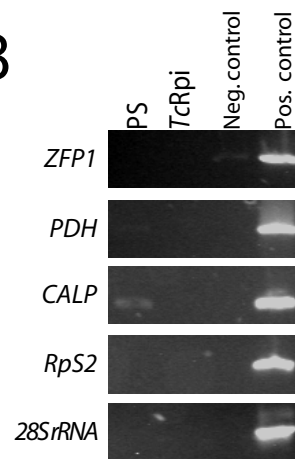

Supplement: Additional file 1 — Immunoprecipitation control and RT-PCR. As an internal control, IP and RT-PCR reactions were performed using a non-related antibody, anti-TcRpi. The same conditions and amplification cycles were used. A) Western blot employing anti-TcRpi in immunoprecipitated samples. The position of the bands corresponding to each protein and to the heavy chain (hc) of antibodies is indicated with an arrow on the left side of the panel. Molecular mass protein standards Dalton Mark VII-L™ are indicated on the right side. B) PCR amplification using specific sets of primers to confirm the absence of some identified transcripts for TcUBP1 and TcRBP3. PS, preimmune serum; TcRpi, T. cruzi Ribose 5-phosphate isomerase Type B. [file 1471-2199-9-107-S1.pdf]
