# Supplementary material for: Functionally related transcripts have common RNA motifs for specific RNA-binding proteins in trypanosomes
Source: BMC Mol Biol. 2008 Dec 8;9:107. doi: 10.1186/1471-2199-9-107 (PMC2637893; doi:10.1186/1471-2199-9-107)
Supplement: Additional file 2 — List of database targets harboring UBP1m. Sequences obtained from dbEST bearing UBP1m were filtered using the annotation file provided by TIGR and manually classified into functional categories. N, number of sequences found. [file 1471-2199-9-107-S2.doc]

**Additional file 2.** List of database targets harboring UBP1m.

| GenBank Acc. Num. | TIGR Description | N |
| --- | --- | --- |
|  |  |  |
| ***Metabolism*** | |  |
| AA835614 | weakly similar to UP|Q9K6G2 (Q9K6G2) Ribose 5-phosphate epimerase (Pentose phosphate), partial (63%) | 3 |
| AA532145 | similar to UP|Q34765 (Q34765) NADH dehydrogenase subunit 1 (Fragment), partial (42%) | 2 |
| AI069753 | similar to UP|Q70NW5 (Q70NW5) NADH dehydrogenase subunit 5, partial (6%) | 4 |
| CF889335 | UP|Q33570 (Q33570) ATPase subunit 6 | 1 |
| AA882644 | homologue to UP|Q8T303 (Q8T303) Tcc1j12.2, partial (48%) | 11 |
| AA926619 | homologue to PDB|1II2_A.0|17942708|1II2_A Chain A, Crystal Structure Of Phosphoenolpyruvate Carboxykinase (Pepck) From Trypanosoma Cruzi. {Trypanosoma cruzi;} , partial (19%) | 3 |
| AA952345 | weakly similar to UP|GNPI_MOUSE (O88958) Glucosamine-6-phosphate isomerase (Glucosamine-6-phosphate deaminase) (GNPDA) (GlcN6P deaminase) (Oscillin) , partial (39%) | 1 |
| AA525713 | similar to UP|NAGB_PASMU (Q9CMF4) Glucosamine-6-phosphate deaminase (Glucosamine-6-phosphate isomerase) (GNPDA) (GlcN6P deaminase) , partial (26%) | 1 |
| AA890926 | UP|Q8ST54 (Q8ST54) Hexokinase, complete | 2 |
| AA441761 | similar to UP|Q9T2V5 (Q9T2V5) Cytochrome C oxidase subunit 5 (Fragment), complete | 1 |
| AA908108 | UP|O48239 (O48239) Cytochrome b (Fragment), partial (7%) | 10 |
| AI668080 | similar to UP|COX1_CRION (P98003) Cytochrome c oxidase polypeptide I (Fragment) , partial (5%) | 1 |
| AI037804 | UP|O00821 (O00821) Clone TcCE5-7-1, complete | 1 |
| CF888137 | Trypanosoma cruzi DGC3 mRNA, partial sequence | 2 |
| AI667951 | weakly similar to UP|Q7U8E0 (Q7U8E0) Predicted alpha/beta hydrolase superfamily protein, partial (6%) | 1 |
| AA882599 | UP|MIP_TRYCR (Q09734) Macrophage infectivity potentiator precursor (Peptidyl-prolyl cis-trans isomerase) (PPIase) (Rotamase) , complete | 2 |
| AI046038 | weakly similar to UP|Q8H5F0 (Q8H5F0) Betaine aldehyde dehydrogenase-like, partial (7%) | 1 |
| AI050140 | similar to UP|Q27680 (Q27680) Phosphoribosylpyrophosphate synthetase , partial (16%) | 1 |
| AI026578 | similar to UP|METK_LEIIN (O43938) S-adenosylmethionine synthetase (Methionine adenosyltransferase) (AdoMet synthetase) , partial (20%) | 1 |
| AA952566 | homologue to UP|Q7YUF0 (Q7YUF0) S-adenosylhomocysteine hydrolase , partial (59%) | 2 |
| AI080794 | weakly similar to UP|DIMH_DROME (Q9VAQ5) Probable dimethyladenosine transferase (S-adenosylmethionine-6-N',N'-adenosyl(rRNA) dimethyltransferase) (18S rRNA dimethylase) , partial (47%) | 1 |
| AI069754 | UP|O61101 (O61101) TcC31.26, partial (20%) | 3 |
| AA556111 | similar to UP|Q7YW97 (Q7YW97) L-threonine 3-dehydrogenase , partial (94%) | 2 |
| AA952480 | similar to UP|RPE_YEAST (P46969) Ribulose-phosphate 3-epimerase (Pentose-5-phosphate 3-epimerase) (PPE) (RPE) , partial (7%) | 3 |
| AA433314 | UP|Q836C7 (Q836C7) Hydrolase, haloacid dehalogenase family, partial (5%) | 1 |
| AI069696 | UP|Q7YWE5 (Q7YWE5) Methylthioadenosine phosphorylase , complete | 1 |
| AA958038 | similar to UP|Q7VN25 (Q7VN25) Mannose-specific phosphotransferase system IID component, partial (5%) | 1 |
| AA952497 | weakly similar to GB|BAA01504.1|220838|RATPDCE2 dihydrolipoamide acetyltransferase {Rattus norvegicus;} , partial (23%) | 1 |
| AA556071 | weakly similar to GB|BAB08924.1|9758516|AB016882 carnitine/acylcarnitine translocase-like protein {Arabidopsis thaliana;} , partial (13%) | 1 |
| AI057698 | weakly similar to UP|Q835L4 (Q835L4) Hydroxymethylglutaryl-CoA synthase, partial (9%) | 2 |
| AA676135 | weakly similar to UP|Q9I9K6 (Q9I9K6) Prolyl isomerase Pin1, partial (38%) | 1 |
|  |  |  |
| ***Cell division and DNA synthesis*** | |  |
| AA532124 | NUP-1 [Trypanosoma cruzi] | 12 |
| AI069780 | weakly similar to UP|Q9U1E0 (Q9U1E0) DNA replication licensing factor (CDC47 homolog), partial (8%) | 1 |
| AA556017 | UP|H2A_TRYCR (P35066) Histone H2A, complete | 3 |
| AI057727 | similar to UP|Q27056 (Q27056) Spliced leader mRNA (pSLc3) from procyclic stage. (Fragment), complete | 1 |
| AA676155 | weakly similar to UP|Q7YW24 (Q7YW24) Retrotransposon hot spot protein RHS1, partial (4%) | 1 |
| AI562289 | similar to UP|Q867G5 (Q867G5) Histone H3 variant, partial (69%) | 5 |
| AA882496 | similar to UP|Q8PFK2 (Q8PFK2) Histone H1 homolog, partial (6%) | 1 |
| AA960728 | UP|Q9GQL7 (Q9GQL7) Cyclin 6, complete | 1 |
| AA532115 | similar to UP|Q9U9A5 (Q9U9A5) Histone H4, complete | 2 |
| AI066184 | weakly similar to UP|RPBX_SCHPO (O13877) DNA-directed RNA polymerases I, II, and III 8.3 kDa polypeptide (ABC10-beta) , partial (87%) | 2 |
| AA926607 | similar to UP|Q9U701 (Q9U701) Histone H2A unit A, partial (49%) | 2 |
| AA399704 | homologue to UP|H2B_TRYCR (P27795) Histone H2B, complete | 2 |
| AI066379 | homologue to UP|Q9Y026 (Q9Y026) NL1Tc (Fragment), partial (38%) | 1 |
| AI035177 | homologue to UP|Q9JM93 (Q9JM93) SRp25 nuclear protein (ADP-ribosylation factor-like 6 interacting protein 4), partial (5%) | 1 |
| AI007363 | similar to UP|Q9WUG8 (Q9WUG8) ARL-6 interacting protein-4 (Fragment), partial (15%) | 2 |
|  |  |  |
| ***Transcription*** | |  |
| CB924100 | homologue to UP|P93392 (P93392) S25-XP1 DNA binding protein, partial (5%) | 2 |
| AI667924 | similar to UP|Q9W4J8 (Q9W4J8) CG17592-PB, partial (4%) | 1 |
| AA908077 | weakly similar to UP|ENL_HUMAN (Q03111) ENL protein, partial (4%) | 2 |
| AI035164 | similar to UP|Q8T052 (Q8T052) LD27895p (CG18009-PD) (Cg18009-pa), partial (3%) | 1 |
| AI035088 | similar to UP|Q86NM5 (Q86NM5) SD06504p, partial (3%) | 1 |
| AA676130 | weakly similar to GB|BAA14006.1|1731809|D89667 c-myc binding protein {Homo sapiens;} , partial (16%) | 1 |
| AA738523 | weakly similar to UP|ATF5_HUMAN (Q9Y2D1) Cyclic-AMP-dependent transcription factor ATF-5 (Activating transcription factor 5) (Transcription factor ATFx), partial (5%) | 1 |
|  |  |  |
|  | RNA processing, protein synthesis and degradation |  |
| AA556058 | homologue to UP|Q61402 (Q61402) Gcap1 protein (Fragment), partial (26%) | 9 |
| AI066272 | similar to UP|Q9BHM3 (Q9BHM3) Cyclophilin-RNA interacting protein, partial (3%) | 1 |
| AI035065 | similar to UP|Q99942 (Q99942) G16 (HsRma1) (Ring finger protein 5), partial (8%) | 2 |
| AA676098 | weakly similar to GB|AAH26525.1|20072952|BC026525 Auh protein {Mus musculus;} , partial (10%) | 5 |
| AA676151 | weakly similar to UP|Q9GYZ6 (Q9GYZ6) Sm-G, complete | 1 |
| AI110429 | weakly similar to PIR|T00956|T00956 translation initiation factor eIF-2 gamma chain F20D22.6 - Arabidopsis thaliana {Arabidopsis thaliana;} , partial (11%) | 1 |
| AA882885 | similar to UP|Q72PP5 (Q72PP5) Prepilin leader peptidase, partial (8%) | 1 |
| AA426689 | homologue to UP|Q8T2Y4 (Q8T2Y4) Tcc1l8.8, partial (97%) | 2 |
| AA676110 | weakly similar to UP|Q9N8P8 (Q9N8P8) Calpain-like protein, possible, partial (37%) | 3 |
| AA882994 | similar to UP|Q9BML1 (Q9BML1) ATP-dependent zinc metallopeptidase-like protein, partial (30%) | 3 |
| AA952746 | homologue to UP|Q849C5 (Q849C5) Caspase-9 long chain, partial (18%) | 2 |
| AI622953 | homologue to UP|O61066 (O61066) Cathepsin B-like protease precursor, partial (9%) | 4 |
| AI057735 | homologue to UP|PSA2_TRYBB (Q9U793) Proteasome subunit alpha type 2 (20S proteasome subunit alpha-2) , partial (94%) | 1 |
| AW325103 | UP|PSA1_TRYCR (P92188) Proteasome subunit alpha type 1 (Proteasome 29 kDa subunit) (TCPR29) , complete | 1 |
| CF887947 | similar to UP|Q7RT72 (Q7RT72) Ubiquitin c-terminal extension protein UBIcep86, partial (9%) | 4 |
| AA676053 | homologue to UP|Q9GNC1 (Q9GNC1) Probable ubiquitin-conjugating enzyme e2-17 kDa, partial (17%) | 2 |
| AA882876 | similar to UP|Q963A7 (Q963A7) Co-chaperonin CPN10, partial (98%) | 11 |
| AI077144 | similar to UP|Q08505 (Q08505) P23-like protein (Fragment), partial (17%) | 2 |
| AA676179 | homologue to emb|V01390.1|TBREP3 Trypanosoma brucei kinetoplast DNA maxicircle fragment encoding two very small ribosomal RNAs, a 12S rRNA homologous to E.coli 23S rRNA and a 9S rRNA homologous to 16S rRNA, partial (7%) | 1 |
| AI046117 | homologue to gb|L22334.1|TRBS3RRBN Trypanosoma cruzi 5.8S ribosomal RNA, internal transcribed spacers 1-7 (ITS1-ITS7), and 28S ribosomal RNA, partial (12%) | 14 |
| AI035057 | UP|Q9GZC7 (Q9GZC7) RNA binding protein RGGm, complete | 1 |
| AI066204 | similar to UP|Q9NDA3 (Q9NDA3) 20S proteasome alpha 7 subunit, partial (43%) | 1 |
|  |  |  |
| ***Ribosomal proteins*** | |  |
| AI005689 | weakly similar to UP|RS18_SCHPO (O94754) 40S ribosomal protein S18, partial (88%) | 1 |
| AI057780 | weakly similar to UP|Q86QR6 (Q86QR6) Ribosomal protein L39, partial (96%) | 2 |
| AA738524 | homologue to UP|RLA1_TRYCR (P26643) 60S acidic ribosomal protein P1, partial (94%) | 12 |
| AI053319 | homologue to UP|RLA0_TRYCR (P26796) 60S acidic ribosomal protein P0, partial (58%) | 1 |
| AA867960 | similar to UP|RL29_DROME (Q24154) 60S ribosomal protein L29 (L43), partial (33%) | 2 |
| AA556143 | similar to UP|Q9N9V4 (Q9N9V4) Ribosomal protein S25, partial (79%) | 1 |
| AA426672 | similar to UP|RS6_LEIMA (Q9NE83) 40S ribosomal protein S6, partial (91%) | 2 |
| AA867944 | similar to UP|RS8_LEIMA (P25204) 40S ribosomal protein S8, partial (86%) | 1 |
| AA556056 | UP|RLA2_TRYCR (P23632) 60S acidic ribosomal protein P2-A (P) (P-JL5) (L12E), complete | 7 |
| AA952714 | weakly similar to UP|RL22_HUMAN (P35268) 60S ribosomal protein L22 (Epstein-Barr virus small RNA associated protein) (EBER associated protein) (EAP) (Heparin binding protein HBp15), partial (34%) | 1 |
| AA676024 | weakly similar to GB|AAK95160.1|15293935|AF401588 ribosomal protein L34 {Ictalurus punctatus;} , partial (45%) | 4 |
| AA676047 | similar to GB|AAG32534.1|11245770|AY007805 ribosomal protein L36 {Dictyostelium discoideum;} , partial (49%) | 2 |
| AI563032 | weakly similar to UP|Q8SYG0 (Q8SYG0) RE63456p, partial (16%) | 1 |
| CF888935 | homologue to UP|Q9XZ65 (Q9XZ65) Laminin receptor-like protein/ p40 ribosome associated-like protein, partial (37%) | 1 |
|  |  |  |
| ***Glycoproteins*** | |  |
| AI050176 | UP|Q9NG31 (Q9NG31) GP63-3 protein, partial (50%) | 1 |
| AA433306 | homologue to UP|Q9NIQ8 (Q9NIQ8) Mucin-like protein, complete | 11 |
| AI069905 | UP|Q26856 (Q26856) Amastin, complete | 3 |
| AI080898 | UP|GP85_TRYCR (Q03877) 85 kDa surface antigen precursor, partial (94%) | 1 |
| AI562296 | UP|Q16861 (Q16861) Super cysteine rich protein (Fragment), partial (30%) | 1 |
| AI077195 | UP|Q26852 (Q26852) Surface glycoprotein, complete | 1 |
| AA882656 | similar to UP|Q26890 (Q26890) Surface antigen, partial (11%) | 3 |
| AA882829 | UP|Q26946 (Q26946) P-glycoprotein, complete | 1 |
| AA676138 | similar to UP|Q7M3R7 (Q7M3R7) Repetitive protein antigen 101 (Fragment), complete | 2 |
| AA875703 | weakly similar to UP|Q7T390 (Q7T390) Glycoprotein, synaptic 2, partial (15%) | 3 |
| AA952342 | similar to UP|Q86DL7 (Q86DL7) Surface protein-2, partial (3%) | 1 |
| AA676001 | homologue to UP|Q8QRV0 (Q8QRV0) Glycoprotein US9, partial (6%) | 1 |
| AI069907 | UP|Q9U7F3 (Q9U7F3) Stage-specific surface glycoprotein gp82 (Fragment), complete | 1 |
| AA882535 | Trypanosoma cruzi 11o14 mucin-like protein (SMUG gene), 3' UTR | 2 |
| AA882571 | homologue to UP|Q03625 (Q03625) Sialidase (Fragment) , partial (3%) | 2 |
| AI035222 | similar to UP|Q24007 (Q24007) Gp150 protein (CG5820-PA) (Cg5820-pb) (RE46351p), partial (3%) | 1 |
| AA882611 | homologue to UP|Q8T2Z9 (Q8T2Z9) Tcc1j12.6, partial (49%) | 1 |
|  |  |  |
| ***Stress and signaling*** | |  |
| AA952423 | UP|O96507 (O96507) Arginine kinase, complete | 3 |
| AI053291 | similar to UP|Q72DX6 (Q72DX6) Sensor histidine kinase, partial (4%) | 1 |
| AI075585 | similar to UP|Q94JZ6 (Q94JZ6) Protein kinase-like protein (At3g24600), partial (3%) | 1 |
| AI110420 | homologue to UP|Q87A74 (Q87A74) Phosphatidylglycerophosphatase B, partial (5%) | 1 |
| AI035170 | weakly similar to UP|CALB_SCHPO (Q9UU93) Calcineurin B subunit (Protein phosphatase 2B regulatory subunit) (Calcineurin regulatory subunit), partial (63%) | 1 |
| AI046077 | similar to UP|Q7DLS8 (Q7DLS8) PRL1 protein (Fragment), partial (5%) | 2 |
| AI110364 | weakly similar to GB|AAH09337.2|14424624|BC009337 MLL4 protein {Homo sapiens;} , partial (3%) | 1 |
| AA426682 | UP|PTCA_HUMAN (Q14761) Protein tyrosine phosphatase receptor type C-associated protein (PTPRC-associated protein) (CD45-associated protein) (CD45-AP) (Lymphocyte phosphatase-associated phosphoprotein), partial (5%) | 2 |
| AI065223 | weakly similar to UP|O62367 (O62367) C. elegans SRA-23 protein (Corresponding sequence T06G6.1), partial (6%) | 1 |
| AI066140 | homologue to UP|Q95VS9 (Q95VS9) Centrin, complete | 2 |
| AA675965 | homologue to UP|Q8T2V8 (Q8T2V8) Tcc1i14-2.7, partial (34%) | 2 |
| AA952502 | similar to UP|YPT6_YEAST (Q99260) GTP-binding protein YPT6, partial (11%) | 1 |
| AA676112 | similar to UP|Q25325 (Q25325) Heat shock protein 70-related protein, partial (25%) | 2 |
| CF887968 | UP|CALM_TRYCR (P18061) Calmodulin (CaM), complete | 1 |
|  |  |  |
| ***Transport*** | |  |
| AA866529 | weakly similar to UP|KDD4_HUMAN (Q8WVF5) Potassium channel tetramerisation domain containing protein 4, partial (18%) | 2 |
| AA426668 | homologue to UP|Q26858 (Q26858) Hexose transporter, complete | 1 |
| AA676060 | weakly similar to UP|Q8WPU7 (Q8WPU7) Probable biopterin transporter (Esag10), partial (29%) | 2 |
| AA532141 | weakly similar to UP|S6G1_ARATH (Q9SW34) Protein transport protein SEC61 gamma-1 subunit, partial (70%) | 5 |
| AW324877 | weakly similar to UP|Q7CSQ0 (Q7CSQ0) AGR_L_2595p, partial (9%) | 1 |
| AA556117 | homologue to UP|Q7TQ10 (Q7TQ10) Aa1027, partial (10%) | 2 |
| AI077243 | weakly similar to UP|Q7QL66 (Q7QL66) AgCP3069 (Fragment), partial (5%) | 2 |
|  |  |  |
|  | Biogenesis, molecular motors and cell organization |  |
| AA952466 | UP|Q7YSU1 (Q7YSU1) Actin 1 (Actin 2) (Actin 3), complete | 1 |
| CF887970 | homologue to UP|Q7Z1E2 (Q7Z1E2) Clathrin assembly protein AP19-like protein, partial (91%) | 1 |
| AI043471 | homologue to UP|Q81113 (Q81113) Core protein precursor, partial (6%) | 1 |
| CF888390 | similar to UP|Q874C3 (Q874C3) Longevity-assurance protein, partial (5%) | 2 |
| AI077073 | UP|O46353 (O46353) Paraflagellar rod component Par4, complete | 1 |
| AI035089 | similar to UP|Q6Y235 (Q6Y235) High mobility group protein, partial (6%) | 1 |
| AA426679 | UP|Q8T9X5 (Q8T9X5) Alpha tubulin, complete | 1 |
| AA441774 | weakly similar to UP|Q7TPK7 (Q7TPK7) Ac2-048, partial (8%) | 3 |
| AA960734 | weakly similar to UP|Q7ZVW0 (Q7ZVW0) RAB28, member RAS oncogene family, partial (23%) | 1 |
| AI035015 | similar to UP|Q26694 (Q26694) Ras-related protein RAB-5, partial (78%) | 1 |
| AA926483 | similar to UP|Q9XY95 (Q9XY95) Neurotrophin, partial (7%) | 1 |
| AA532106 | similar to UP|O74112 (O74112) Krev-1 protein, partial (6%) | 1 |
| AA952399 | weakly similar to UP|Q9VBU6 (Q9VBU6) CG11857-PA (RE24638p), partial (27%) | 2 |
| AI069598 | weakly similar to GB|AAF64304.1|7582386|AF249273 Bcl-2-associated transcription factor short form {Homo sapiens;} , partial (3%) | 1 |
| CF888338 | similar to GB|AAC34212.1|3478639|AC005545 delta-adaptin, partial CDS {Homo sapiens;} , partial (3%) | 4 |
| AW329952 | similar to PRF|1405285A.0|225989|1405285A thyroglobulin. {Bos taurus;} , partial (5%) | 3 |
| AI562371 | similar to UP|Q7TPA6 (Q7TPA6) Ab1-042, partial (12%) | 1 |
| AA908101 | similar to UP|Q8NG09 (Q8NG09) LIR-D1, partial (3%) | 3 |
| AA958050 | weakly similar to UP|Q8S9K1 (Q8S9K1) At1g23880/T23E23_8, partial (4%) | 2 |
| AA882958 | similar to UP|SEP3_MOUSE (Q9Z1S5) Neuronal-specific septin 3, partial (4%) | 2 |
| AI717809 | weakly similar to GB|AAM10380.1|20147333|AY093756 At2g27260/F12K2.16 {Arabidopsis thaliana;} , partial (8%) | 1 |
|  |  |  |
| ***Unknown*** | |  |
| CF888383 | homologue to UP|Q69566 (Q69566) U88, partial (5%) | 2 |
| AI046091 | homologue to UP|Q7PJ70 (Q7PJ70) ENSANGP00000023822 (Fragment), partial (11%) | 1 |
| AI007441 | homologue to UP|Q7PI42 (Q7PI42) ENSANGP00000024316 (Fragment), partial (5%) | 2 |
| AI667882 | similar to UP|Q7RXZ6 (Q7RXZ6) Predicted protein, partial (3%) | 4 |
| AA952375 | weakly similar to PIR|E64819|E64819 ybiU protein - Escherichia coli (strain K-12) {Escherichia coli;} , partial (25%) | 3 |
| AI026473 | UP|Q80WG6 (Q80WG6) UROP11-110, partial (10%) | 2 |
| CB923991 | cDNA52 product | 1 |
| AI077275 | GB|AAQ88431.1|37181043|AY380798 Arzc-1 {Mus musculus;} , partial (12%) | 2 |
| AA890770 | similar to UP|Q37909 (Q37909) ORF2 protein, partial (6%) | 3 |
| AA926432 | similar to UP|Q76ND2 (Q76ND2) ORF41c, partial (46%) | 2 |
| CF888314 | similar to UP|Q7PKG0 (Q7PKG0) ENSANGP00000024462 (Fragment), partial (43%) | 14 |
| AA866512 | similar to UP|Q7PWX9 (Q7PWX9) EbiP4168 (Fragment), partial (50%) | 2 |
| AA532156 | UP|Q8C8C9 (Q8C8C9) Mus musculus 10 days neonate cerebellum cDNA, RIKEN full-length enriched library, clone:B930063N02 product:speckle-type POZ protein, full insert sequence, partial (10%) | 9 |
| CB964169 | homologue to UP|Q8C372 (Q8C372) Mus musculus 15 days embryo head cDNA, RIKEN full-length enriched library, clone:D930047C10 product:unclassifiable, full insert sequence. (Fragment), partial (5%) | 2 |
| CF888834 | similar to UP|Q8BUX8 (Q8BUX8) Mus musculus 16 days embryo head cDNA, RIKEN full-length enriched library, clone:C130083B15 product:EYES ABSENT HOMOLOG 4, full insert sequence, partial (17%) | 1 |
| AA958032 | similar to GB|CAA86883.1|854495|SCCHXIV43 orf6 {Saccharomyces cerevisiae;} , partial (11%) | 2 |
| AA532095 | similar to UP|Q7X1H1 (Q7X1H1) Lfe142p3, partial (37%) | 4 |
| AA441774 | weakly similar to UP|Q7TPK7 (Q7TPK7) Ac2-048, partial (8%) | 3 |
| AA676065 | similar to UP|Q8LLC2 (Q8LLC2) Hv711N16.16 (Fragment), partial (7%) | 3 |
| AI053269 | homologue to UP|Q9VUE1 (Q9VUE1) CG13482-PA, partial (12%) | 1 |
| AW330011 | similar to GB|AAD56725.1|5931961|AF124727 acinusS {Homo sapiens;} , partial (4%) | 2 |
| AA882897 | similar to GB|AAD55332.1|5901535|AF089873 protamine P1 {Sminthopsis bindi;} , partial (29%) | 2 |
| AA556062 | N9 protein | 1 |
| AI026557 | homologue to UP|Q95RT0 (Q95RT0) LD12764p, partial (9%) | 1 |
| AI057915 | similar to UP|O57150 (O57150) H88, partial (68%) | 1 |
| CF888014 | similar to UP|Q8GBE6 (Q8GBE6) Yts1D protein, partial (3%) | 3 |
| AI077208 | similar to UP|Q8IPR6 (Q8IPR6) CG15593-PB, partial (4%) | 1 |
| AI046261 | similar to UP|Q8I727 (Q8I727) TcC31.32, partial (13%) | 1 |
| AI667940 | similar to UP|Q96DG4 (Q96DG4) LOC124245 protein (Fragment), partial (3%) | 1 |
| AA908112 | similar to UP|Q99KF9 (Q99KF9) Lrpprc protein, partial (32%) | 4 |
| CB964194 | similar to UP|Q9HBL2 (Q9HBL2) HT018, partial (6%) | 1 |
| AI026544 | T.cruzi mRNA with SIRE sequence, clone o16 | 1 |
| AA441745 | Trypanosoma cruzi clone SAE.p1.87 SIRE repeat region | 2 |
| AI065173 | Trypanosoma cruzi SIRE repeat region | 2 |
| AI053275 | UP|Q6QI50 (Q6QI50) LRRGT00158, partial (5%) | 1 |
| AA433311 | UP|Q6TXI9 (Q6TXI9) LRRGT00010, partial (12%) | 1 |
| AA958185 | UP|Q7D1G4 (Q7D1G4) AGR_C_785p, partial (12%) | 1 |
| AI046258 | UP|Q7PHQ2 (Q7PHQ2) ENSANGP00000022785 (Fragment), partial (8%) | 1 |
| AI035004 | weakly similar to UP|Q757M5 (Q757M5) AEL011Wp, partial (4%) | 1 |
| AI035190 | weakly similar to UP|Q7PZR5 (Q7PZR5) AgCP9442, partial (27%) | 1 |

Sequences obtained from dbEST bearing m1 were filtered using the annotation file provided by TIGR and manually classified into functional categories. N, number of sequences found.
