# Supplementary material for: Functionally related transcripts have common RNA motifs for specific RNA-binding proteins in trypanosomes
Source: BMC Mol Biol. 2008 Dec 8;9:107. doi: 10.1186/1471-2199-9-107 (PMC2637893; doi:10.1186/1471-2199-9-107)
Supplement: Additional file 3 — List of database targets bearing RBP3m. Sequences obtained from dbEST bearing RBP3m were filtered using the annotation file provided by TIGR and manually classified into functional categories. N, number of sequences found. [file 1471-2199-9-107-S3.doc]

**Additional file 3.** List of database targets bearing RBP3m.

| GenBank Acc. Num. | TIGR Description | N | |
| --- | --- | --- | --- |
|  |  |  |  |
| ***Metabolism*** |  |  |  |
| AA926619 | homologue to PDB|1II2_A.0|17942708|1II2_A Chain A, Crystal Structure Of Phosphoenolpyruvate Carboxykinase (Pepck) From Trypanosoma Cruzi. {Trypanosoma cruzi;} , partial (19%) | 2 |  |
| AA890874 | UP|Q6JCT3 (Q6JCT3) Cytochrome b (Fragment), partial (6%) | 3 |  |
| AI050125 | UP|TYTR_TRYCR (P28593) Trypanothione reductase (TR) (N(1),N(8)-bis(glutathionyl)spermidine reductase) , complete | 3 |  |
| AA882512 | weakly similar to UP|Q6W203 (Q6W203) Electron transfer flavoprotein beta-subunit, partial (55%) | 1 |  |
| AI066324 | UP|Q8T1Q1 (Q8T1Q1) Tcc44h21-2.8, partial (56%) | 2 |  |
|  |  |  |  |
|  | Cell division and DNA synthesis |  |  |
| AI667980 | similar to UP|Q6FL10 (Q6FL10) Strain CBS138 chromosome L complete sequence, partial (4%) | 2 |  |
| AA556017 | UP|H2A_TRYCR (P35066) Histone H2A, complete | 2 |  |
|  |  |  |  |
|  | RNA processing, protein synthesis and degradation |  |  |
| CF888231 | similar to UP|Q8VX74 (Q8VX74) Glycine-rich RNA-binding protein, partial (11%) | 2 |  |
| BF317572 | similar to UP|Q8R0K1 (Q8R0K1) Smarca4 protein (Fragment), partial (3%) | 1 |  |
| CF888116 | similar to UP|Q9SP08 (Q9SP08) Exonuclease RRP41, partial (12%) | 1 |  |
| CB964094 | weakly similar to UP|O01418 (O01418) Gag protein, partial (3%) | 1 |  |
| AA426680 | UP|O00819 (O00819) Elongation factor 1-alpha, complete | 1 |  |
| CF888952 | UP|Q6T430 (Q6T430) MP67, complete | 1 |  |
| AI005691 | homologue to gb|L22334.1|TRBS3RRBN Trypanosoma cruzi 5.8S ribosomal RNA, internal transcribed spacers 1-7 (ITS1-ITS7), and 28S ribosomal RNA, partial (4%) | 1 |  |
|  |  |  |  |
| ***Ribosomal proteins*** | |  |  |
| AA676105 | homologue to UP|RL4_TRYBB (P49669) 60S ribosomal protein L4 (L1), partial (26%) | 1 |  |
| AI021826 | similar to UP|Q868B1 (Q868B1) 40S ribosomal protein S5A (40S ribosomal protein S5B), complete | 10 |  |
| AI035226 | weakly similar to UP|Q90YP6 (Q90YP6) 40S ribosomal protein S27-1, partial (92%) | 1 |  |
| AA426703 | weakly similar to UP|Q90YW6 (Q90YW6) Ribosomal protein L5a, partial (69%) | 2 |  |
| AI007411 | UP|Q9BMP1 (Q9BMP1) Ribosomal protein L19-like protein, complete | 7 |  |
| AA867960 | similar to UP|RL29_DROME (Q24154) 60S ribosomal protein L29 (L43), partial (33%) | 2 |  |
| AA426672 | similar to UP|RS6_LEIMA (Q9NE83) 40S ribosomal protein S6, partial (91%) | 10 |  |
| AA926585 | similar to UP|Q9N8L1 (Q9N8L1) 40S ribosomal protein S11, probable, partial (51%) | 2 |  |
| AA399706 | similar to UP|Q9NGK2 (Q9NGK2) 40S ribosomal protein S24e, complete | 2 |  |
|  |  |  |  |
| ***Glycoproteins*** | |  |  |
| AW325115 | UP|Q9M7N4 (Q9M7N4) MFP1 attachment factor 1, partial (7%) | 1 |  |
| AA525734 | UP|Q9NIQ3 (Q9NIQ3) Mucin-like protein, partial (91%) | 4 |  |
| AA556081 | weakly similar to UP|Q7M2Y2 (Q7M2Y2) Tumor-specific transplantation antigen P198 homolog p23 (Fragment), partial (48%) | 3 |  |
| BF146284 | UP|Q9GPB0 (Q9GPB0) Antigen 38, complete | 2 |  |
| AA882582 | UP|Q26873 (Q26873) Tuzin, complete | 1 |  |
| CF889037 | similar to UP|Q41645 (Q41645) Extensin (Fragment), partial (7%) | 4 |  |
|  |  |  |  |
|  |  |  |  |
| ***Stress and signaling*** | |  |  |
| AA532125 | homologue to UP|EFA2_HUMAN (O43921) Ephrin-A2 precursor (EPH-related receptor tyrosine kinase ligand 6) (LERK-6) (HEK7-ligand) (HEK7-L), partial (7%) | 1 |  |
| AI053291 | similar to UP|Q72DX6 (Q72DX6) Sensor histidine kinase, partial (4%) | 1 |  |
| AA676112 | similar to UP|Q25325 (Q25325) Heat shock protein 70-related protein, partial (25%) | 1 |  |
|  |  |  |  |
|  |  |  |  |
| ***Transport*** | |  |  |
| BF299424 | similar to UP|Q9RWV0 (Q9RWV0) Amino acid ABC transporter, permease protein, partial (8%) | 3 |  |
| AI077243 | weakly similar to UP|Q7QL66 (Q7QL66) AgCP3069 (Fragment), partial (5%) | 3 |  |
|  |  |  |  |
| ***Biogenesis, molecular motors and cell organization*** | |  |  |
| AA441774 | weakly similar to UP|Q7TPK7 (Q7TPK7) Ac2-048, partial (8%) | 1 |  |
| AI622932 | weakly similar to UP|Q8PX92 (Q8PX92) Chemotaxis protein CheW, partial (16%) | 2 |  |
| AA926483 | similar to UP|Q9XY95 (Q9XY95) Neurotrophin, partial (7%) | 1 |  |
| AA532189 | UP|AGNX_APLSP (P12284) Neuroactive polyprotein R15-1 precursor [Contains: R15 alpha-2 peptide; R15 beta peptide; R15 gamma peptide], partial (8%) | 1 |  |
| AA433378 | similar to UP|Q40445 (Q40445) Tumor-related protein (Fragment), partial (19%) | 3 |  |
| AI057723 | similar to UP|Q8MKD9 (Q8MKD9) LPS-induced TNF-alpha factor, partial (6%) | 2 |  |
| AA426696 | UP|Q8STF3 (Q8STF3) Beta tubulin 1.9 (Beta tubulin 2.3), complete | 1 |  |
|  |  |  |  |
| ***Unknown*** | |  |  |
| AA890906 | weakly similar to UP|Q6ZPR1 (Q6ZPR1) MKIAA1430 protein (Fragment), partial (4%) | 1 |  |
| AI034992 | UP|Q03884 (Q03884) S.cerevisiae chromosome IX cosmid 9150, partial (12%) | 1 |  |
| AA952526 | similar to UP|Q8IP68 (Q8IP68) CG31813-PA, partial (8%) | 1 |  |
| AI057895 | weakly similar to UP|Q8QNE4 (Q8QNE4) EsV-1-135, partial (5%) | 3 |  |
| CF888274 | similar to UP|Q6TUE7 (Q6TUE7) LRRGT00097, partial (4%) | 3 |  |
| AI562296 | UP|Q16861 (Q16861) Super cysteine rich protein (Fragment), partial (30%) | 1 |  |

The key is as for Additional file 2.
