# Supplementary material for: Functionally related transcripts have common RNA motifs for specific RNA-binding proteins in trypanosomes
Source: BMC Mol Biol. 2008 Dec 8;9:107. doi: 10.1186/1471-2199-9-107 (PMC2637893; doi:10.1186/1471-2199-9-107)
Supplement: Additional file 4 — Several transcripts have different motifs for either TcUBP1 or TcRBP3. This table contains a list of mRNA target hits bearing the highest four UBP1- and RBP3-motifs. [file 1471-2199-9-107-S4.doc]

**Additional file 4.** List of mRNA target hits bearing the highest four UBP1- and RBP3-motifs.

| Motifs | GenBank Acc. Num. | TIGR Description |
| --- | --- | --- |
| *Tc*UBP1 | AA426668 | homologue to UP|Q26858 (Q26858) Hexose transporter, complete |
|  | CF888028 | homologue to UP|Q7PKG0 (Q7PKG0) ENSANGP00000024462 (Fragment), partial (27%) |
|  | AA676047 | similar to GB|AAG32534.1|11245770|AY007805 ribosomal protein L36 {Dictyostelium discoideum;} , partial (49%) |
|  | AI035015 | similar to UP|Q26694 (Q26694) Ras-related protein RAB-5, partial (78%) |
|  | AI035164 | similar to UP|Q8T052 (Q8T052) LD27895p (CG18009-PD) (Cg18009-pa), partial (3%) |
|  | AA532156 | UP|Q8C8C9 (Q8C8C9) Mus musculus 10 days neonate cerebellum cDNA, RIKEN full-length enriched library, clone:B930063N02 product:speckle-type POZ protein, full insert sequence, partial (10%) |
|  | AA676098 | weakly similar to GB|AAH26525.1|20072952|BC026525 Auh protein {Mus musculus;} , partial (10%) |
| *Tc*RBP3 | CF888343 | homologue to GB|AAF31039.1|6899662|AC005893 ribosomal protein L38 {Leishmania major;} , partial (47%) |
|  | CF888343 | homologue to gb|L22334.1|TRBS3RRBN Trypanosoma cruzi 5.8S ribosomal RNA, internal transcribed spacers 1-7 (ITS1-ITS7), and 28S ribosomal RNA, partial (4%) |
|  | AI005691 | homologue to UP|H2B_TRYCR (P27795) Histone H2B, complete |
|  | AA399704 | similar to UP|O65073 (O65073) Transcription factor BTF3 homolog (Fragment), partial (25%) |
|  | AA676033 | similar to UP|Q8IP68 (Q8IP68) CG31813-PA, partial (8%) |
|  | AA952526 | similar to UP|Q963A7 (Q963A7) Co-chaperonin CPN10, partial (98%) |
|  | AA882876 | similar to UP|Q9BHM3 (Q9BHM3) Cyclophilin-RNA interacting protein, partial (3%) |
|  | AI066272 | similar to UP|Q9HBL2 (Q9HBL2) HT018, partial (6%) |
|  | CB964194 | similar to UP|Q9JM93 (Q9JM93) SRp25 nuclear protein (ADP-ribosylation factor-like 6 interacting protein 4), partial (6%) |
|  | AA926406 | similar to UP|Q9RWV0 (Q9RWV0) Amino acid ABC transporter, permease protein, partial (8%) |
|  | BF299424 | similar to UP|Q9U9A5 (Q9U9A5) Histone H4, complete |
|  | AA532115 | similar to UP|Q9XY95 (Q9XY95) Neurotrophin, partial (7%) |
|  | AA926483 | similar to UP|RL29_DROME (Q24154) 60S ribosomal protein L29 (L43), partial (33%) |
|  | AA867960 | UP|H2A_TRYCR (P35066) Histone H2A, complete |
|  | AA556017 | UP|O00819 (O00819) Elongation factor 1-alpha, complete |
|  | AA426680 | UP|Q03884 (Q03884) S.cerevisiae chromosome IX cosmid 9150, partial (12%) |
|  | AI034992 | UP|Q9NIQ3 (Q9NIQ3) Mucin-like protein, partial (91%) |
|  | AA525734 | weakly similar to UP|ICAL_PIG (P12675) Calpain inhibitor (Calpastatin), partial (3%) |
|  | AA908145 | weakly similar to UP|Q9K6G2 (Q9K6G2) Ribose 5-phosphate epimerase (Pentose phosphate), partial (63%) |
|  | AA835614 | weakly similar to UP|RS10_HUMAN (P46783) 40S ribosomal protein S10, partial (33%) |
