# Supplementary material for: Functionally related transcripts have common RNA motifs for specific RNA-binding proteins in trypanosomes
Source: BMC Mol Biol. 2008 Dec 8;9:107. doi: 10.1186/1471-2199-9-107 (PMC2637893; doi:10.1186/1471-2199-9-107)
Supplement: Additional file 5 — PCR primers. This table contains a list of oligonucleotides used in this work for RT-PCR. [file 1471-2199-9-107-S5.doc]

**Additional file 5.** List of oligonucleotides used in this work for RT-PCR.

| Primer name | Sequence (from 5’ to 3’) |
| --- | --- |
| PDH Fwd | CTCTCTCCGCCCGCTTGACC |
| PDH Rev | GGCGGGCTACGGCAAGAACA |
| Calpain Fwd | GAGGGACCCAACGGACGGAA |
| Calpain Rev | GTCGTCATTGGCTTGCGCGG |
| RpS2 Fwd | CCACACACGATCCCGATGAA |
| RpS2 Rev | TCCGTCTCTGCCCAAAGGTC |
| Parb1 Fwd | GGGTGTGTCTCTCTCTCTCTCTG |
| Parb1 Rev | CCTTCTTTTCTTCCATTTCTTCC |
| MTP Fwd | CCAGCATTTATCTACTCCTCTTTC |
| MTP Rev | CCGCCCCAGGTATCTTTGTACTT |
| RpS8 Fwd | AGAGCACGACGGAGAAGAAGAAG |
| RpS8 Rev | GCGCCCTCCAGCAGTGCACCATC |
| ZFP1 Fwd | CCCCAAACACCCACATTTTCC |
| ZFP1 Rev | CAGTTCTCGAACAACACTCCA |
| RBP3 Fwd | TCCCCCGGGGGAATGTCTGTGGAACGGGGT |
| RBP3 Rev | TCCCCCGGGGAACGACGACCCCCCTCCTC |
| proteasome Fwd | GACGTATTTGACTTTAAGGCAACGG |
| proteasome Rev | AGCGTTCTTGCAGAGTTTCCTCC |
| 28S rRNA Fwd | GTAGTATAGGTGGAAGCCCAAG |
| 28S rRNA Rev | CCAGCTCACGTTCCCTGTCA |
| ZFP1-qPCR Rev | CGATTTAGCGCTCTGTGATGG |
| PDH-qPCR Fwd | GAGGGAAGTGAAATGATGTATTTGTG |
| PDH-qPCR Rev | TCATTGTATCCGCGCTGCT |
| Calpain-qPCR Rev | GCCTGCATTTTGCCTTGC |
| Calpain-qPCR Rev | GCCTGCATTTTGCCTTGC |
| RpS2-qPCR Fwd | TGGTGGATTTTACGGGCATT |
| RpS2-qPCR Rev | ACCATCTCATTCACGAAGAAACC |
| HYPO1 Fwd* | AACAGCAACAAGTACAATTTTAACC |
| HYPO1 Rev* | GCACCAAACTCCTCATAAGCACAAA |
| T7 | GTAATACGACTCACTATAGGGAGA |
| T7-oligo(dT) | GTAATACGACTCACTATAGGGAGA(T)15 |

*Primers also used for transcript synthesis in RNA-binding assay.
