# Supplementary material for: Functionally related transcripts have common RNA motifs for specific RNA-binding proteins in trypanosomes
Source: BMC Mol Biol. 2008 Dec 8;9:107. doi: 10.1186/1471-2199-9-107 (PMC2637893; doi:10.1186/1471-2199-9-107)
Supplement: Additional file 6 — Primers for motifs and deletions. This table contains a list of oligonucleotides used in this work for construction of motifs and deletions. [file 1471-2199-9-107-S6.doc]

**Additional file 6.** List of oligonucleotides used in this work for construction of motifs and deletions.

| Primer name | Sequence (from 5’ to 3’) |
| --- | --- |
| ZFP1m(-) Fwd | aattcAAAAAAAAGAATGAGCGACGTAAATa |
| ZFP1m(-) Rev | agcttATTTACGTCGCTCATTCTTTTTTTTg |
| ZFP1m(+) Fwd | AGTGCACATAACGAAAAAAAAAAAAGAA |
| ZFP1m(+) Rev | AACTCGTACCCAACACTCCAAAATAA |
| ZFP1m Fwd | aattcAAAAAAAAGAATCTGCATATATATATATATATAACATGGGAGCGACGTAAATa |
| ZFP1m Rev | agcttATTTACGTCGCTCCCATGTTATATATATATATATATGCAGATTCTTTTTTTTg |
| ZFP1m mut Fwd | aattcAAAAAAAAGAAGGCGCCCCCCCCCCCCCCCCCCCCCCGCCAGCGACGTAAATa |
| ZFP1m mut Rev | agcttATTTACGTCGCTGGCGGGGGGGGGGGGGGGGGGGGGGCGCCTTCTTTTTTTTg |
| RpS5m(+) Fwd | TGAACGAGGCGATCTACCTAATGTG |
| RpS5m(+) Rev | CGTTCACAATCTCATCGGCAAGACA |
| RPS5m Fwd | aattcGATCTACCTAATGTGCAAGGGTGCCCGCGAGGCTGCTTTCCGCAAAATCACGACa |
| RPS5m Rev | agcttGTCGTGATTTTGCGGAAAGCAGCCTCGCGGGCACCCTTGCACATTAGGTAGATCg |
| RPS5m(-) Fwd | aattcGATCTACCTAAATCACGACa |
| RPS5m(-) Rev | agcttGTCGTGATTTAGGTAGATCg |
| RPS5m mut Fwd | aattcTGAACGAGGCGATCTACCTACCTCAAGACACTGCCTGAGTGTCTTGCCGATGAGATTGTGAACGg |
| RPS5m mut Rev | agcttGTTCACAATCTCATCGGCAAGACACTCAGGCAGTGTCTTGAGGTAGGTAGATCGCCTCGTTCAg |
| PARB1ab Fwd | AATGAAAGGCAAAAAGAAAG |
| PARB1-a Rev | CGAGACACCCCAACTGACAGGA |
| PARB1-b Rev | TTTACTGGAGCACAGTTTCC |

Restriction endonuclease sites (lower case) were generated after annealing of synthetic complementary forward (Fwd) and reverse (Rev) oligonucleotides.
